# Aortic Stiffness in L-NAME Treated C57Bl/6 Mice Displays a Shift From Early Endothelial Dysfunction to Late-Term Vascular Smooth Muscle Cell Dysfunction

**DOI:** 10.3389/fphys.2022.874015

**Published:** 2022-06-16

**Authors:** Sofie De Moudt, Jhana O. Hendrickx, Cédric Neutel, Dorien De Munck, Arthur Leloup, Guido R.Y. De Meyer, Wim Martinet, Paul Fransen

**Affiliations:** Laboratory of Physiopharmacology, University of Antwerp, Antwerp, Belgium

**Keywords:** aortic stiffness, hypertension, calcium, vascular smooth muscle cell, endothelial function (dysfunction), voltage-gated calcium channel, cardiovascular disease

## Abstract

**Introduction and Aims:** Endothelial dysfunction is recognized as a cardiovascular aging hallmark. Administration of nitric oxide synthase blocker N-Ω-Nitro-L-arginine methyl ester hydrochloride (L-NAME) constitutes a well-known small animal model of cardiovascular aging. Despite extensive phenotypic characterization, the exact aortic function changes in L-NAME treated mice are largely unknown. Therefore, this study presents a longitudinal characterization of the aortic reactivity and biomechanical alterations in L-NAME treated C57Bl/6 mice.

**Methods and Results:** Male C57Bl/6 mice were treated with L-NAME (0.5 mg/ml drinking water) for 1, 2, 4, 8, or 16 weeks. Peripheral blood pressure measurement (tail-cuff) and transthoracic echocardiograms were recorded, showing progressive hypertension after 4 weeks of treatment and progressive cardiac hypertrophy after 8–16 weeks of treatment. Aortic stiffness was measured *in vivo* as aortic pulse wave velocity (aPWV, ultrasound) and *ex vivo* as Peterson modulus (E_p_). Aortic reactivity and biomechanics were investigated *ex vivo* in thoracic aortic rings, mounted isometrically or dynamically-stretched in organ bath set-ups. Aortic stiffening was heightened in L-NAME treated mice after all treatment durations, thereby preceding the development of hypertension and cardiac aging. L-NAME treatment doubled the rate of arterial stiffening compared to control mice, and displayed an attenuation of the elevated aortic stiffness at high distending pressure, possibly due to late-term reduction of medial collagen types I, III, and IV content. Remarkably, endothelial dysfunction, measured by acetylcholine concentration-response stimulation in precontracted aortic rings, was only observed after short-term (1–4 weeks) treatment, followed by restoration of endothelial function which coincided with increased phosphorylation of endothelial nitric oxide synthase (S^1177^). In the late-disease phase (8–16 weeks), vascular smooth muscle cell (VSMC) dysfunction developed, including increased contribution of voltage-dependent calcium channels (assessed by inhibition with diltiazem), basal VSMC cytoplasmic calcium loading (assessed by removal of extracellular calcium), and heightened intracellular contractile calcium handling (assessed by measurement of sarcoplasmic reticulum-mediated transient contractions).

**Conclusion:** Arterial stiffness precedes peripheral hypertension and cardiac hypertrophy in chronic L-NAME treated male C57Bl/6 mice. The underlying aortic disease mechanisms underwent a distinct shift from early endothelial dysfunction to late-term VSMC dysfunction, with continued disease progression.

## Introduction

As the primary lining of every blood vessel in the body, the endothelium comprises a crucial interface between blood and tissue, and governs numerous vascular responses (*e.g.,* contractile tone, leukocyte interaction, proliferation). Endothelial cells exert their function by secretion of small molecule, peptide and protein mediators with autocrine and paracrine effects on neighboring cells (Segers et al., 2018). These factors include, among others, prostacyclins, angiotensin II, and endothelin I. The most studied molecule however is nitric oxide (NO), produced by enzymatic activity of endothelial nitric oxide synthase (eNOS), utilizing l-arginine as a substrate and generating NO from its terminal guanidine nitrogen (Atochin and Huang, 2010). NO was the first gaseous molecule ever accepted as a cell signaling mediator, and the discovery of its vasodilatory function was awarded the 1998 Nobel prize of physiology and medicine (Sorelle, 1998).

NO is produced in both basal and stimulated conditions (Leloup A. J. A. et al., 2015), and its importance is highlighted by an abundance in regulatory systems (Zhao et al., 2015). eNOS function is regulated on the transcriptional level (Fulton, 2016), by substrate and cofactor bioavailability (Siragusa and Fleming, 2016), post-translational modifications (*e.g.*, S^1177^ phosphorylation) (Fulton, 2016), dimerization (Alderton et al., 2001), interactions with calcium and calcium-binding proteins (Busse and Mülsch, 1990), and subcellular localization (Bucci et al., 2000; Drab et al., 2001). Furthermore, eNOS is activated by a multitude of stimuli, such as shear stress, acetylcholine, bradykinin, and insulin (Zhao et al., 2015). Finally, endothelial dysfunction - often defined as impaired NO bioavailability - acts as a common player in most cardiovascular (CV) risk factors (Rajendran et al., 2013), underlining the importance of endothelial NO signaling in CV ageing and disease. Such risk factors include hypertension (Panza et al., 1990; Perticone et al., 2001), arterial stiffness (Leloup et al., 2019), smoking (Korneeva and Sirotin, 2017), diabetes (Calver et al., 1992), obesity (Engin, 2017), inflammation (Yang et al., 2016), and oxidative stress (Cai and Harrison, 2000).

Administration of the NOS blocker N-Ω-Nitro-l-arginine methyl ester hydrochloride (L-NAME) constitutes a well-known small animal model of CV disease, characterized by elevated blood pressure and pulse wave velocity (Paulis et al., 2012), increased peripheral resistance (Obst et al., 2004), and reduced cardiac output (Obst et al., 2004). Vascular beds of L-NAME treated mice can still realize endothelium-dependent vasorelaxation, although significantly diminished, which allows for evaluation of potential pharmacological or other interventions. Reduced eNOS expression in aortic tissue was reported after L-NAME treatment (Nagano et al., 2013), along with decreased plasma and urinary NO_x_ levels (Suda et al., 2002). In rats, a marked increase in aortic angiotensin-converting enzyme (ACE) activity was also observed (Korystova et al., 2012). Despite its extensive use in interventional studies, knowledge of the exact changes in aortic function underlying pronounced CV disease in L-NAME treated mice is currently lacking. Therefore, this study presents a longitudinal characterization of temporal CV disease in L-NAME treated mice in relation to aortic reactivity and biomechanical alterations.

## Material and Methods

### Laboratory Animals and Tissue Collection

All animal experiments were approved by the Ethical Committee of the University of Antwerp and were conducted in accordance to the Guide for the Care and Use of Laboratory Animals, published by the National Institutes of Health (NIH Publication No. 85-23; Revised, 1996). All mice were bred and housed in the animal facility of the University of Antwerp, with a 12 h/12 h light-dark cycle and free access to water and standard chow. Male C57Bl/6 mice received NOS blocker N-Ω-Nitro-L-arginine methyl ester hydrochloride (L-NAME, 0.5 mg/ml) continuously through the drinking water, starting at 8 weeks of age, for 1 (n = 12), 2 (n = 10), 4 (n = 10), 8 (n = 11), or 16 weeks (n = 7) in separate treatment groups. For each L-NAME treated group, a non-treated littermate control group (respectively, n = 10, 9, 10, 11, 10) was studied in parallel. The L-NAME solution was refreshed weekly and weighed as a crude measurement of L-NAME intake. At the end of the experiment, mice were euthanatized by perforation of the diaphragm while under deep anesthesia (pentobarbital sodium, 250 mg/kg ip; Sanofi, Belgium). The thoracic aorta was carefully removed and stripped of adherent tissue, to avoid vasoactive influences of perivascular tissue. Next, aortic rings of 2 mm width were cut starting at the diaphragm. Aortic rings were numbered TA0 to TA5 (proximal to distal thoracic aorta). Of these, two segments were used for *ex vivo* isometric reactivity studies (TA3 and TA4), two segments for *ex vivo* assessment of biomechanical aortic properties (TA1 and TA2), one segments was fixed in formaldehyde for histological staining (TA0) and the remaining segment was snap-frozen (TA5). The heart was also isolated and longitudinally cut in halve, randomly assigned for formaldehyde fixation (histology) or snap-freezing.

### *In Vivo* Cardiovascular Measurements

One week before sacrifice, mice underwent CV tests. Conscious peripheral blood pressure and heart rate were measured with a CODA tail-cuff method as previously described (Fransen et al., 2016). In brief, a pressure-volume sensor was attached distally to an occluding cuff to the tail of conscious restrained mice for blood pressure recording. Systolic and diastolic blood pressure were measured on three consecutive days, of which the final measurement was used. Next, transthoracic echocardiograms were acquired in anesthetized mice (1.5%–2.5% isoflurane v/v (Forene, Abbvie)) using high frequency ultrasound (Vevo2100, Visualsonics). Heart rate was maintained at 500 ± 50 bpm and body temperature between 36 and 38°C. M-mode images were obtained for left ventricular (LV) function evaluation on short axis view, including measurement of left-ventricular posterior wall (LVPW) thickness. Fractional shortening (FS), ejection fraction (EF), LV mass and stroke volume (SV) were calculated. On a four-chamber view, diastolic heart function parameters were assessed using pulsed wave Doppler analysis of blood flow through the mitral valve, which allows for measurement of the E wave, A wave, isovolumic relaxation time and deceleration time, and calculation of the E/A ratio. Finally, abdominal aorta pulse wave velocity (aPWV) was measured using the method described by Di Lascio et al. (2014). In short, B-mode images of aortic diameter and pulsed wave Doppler analysis of velocity were acquired and averaged over several cardiac cycles. aPWV was calculated as dV/2 dln(D) (with dV, velocity change; and dln(D), the variation of the natural logarithm of diameter).

### Isometric Reactivity Studies

2-mm aortic rings were mounted between two parallel wire hooks in a 10-ml organ bath containing Krebs-Ringer solution (composition (mM): NaCl 118; KCl 4.7; CaCl_2_ 2.5; KH_2_PO_4_ 1.2; MgSO_4_ 1.2; NaHCO_3_ 25; CaEDTA 0.025; glucose 11.1). The solution was continuously heated to 37°C and aerated with a 95% O_2_/5% CO_2_ gas mixture to maintain a pH of 7.4. In isometric conditions, a preload of 20 mN was applied to approximate normal physiological stretch at a mean blood pressure of 100 mmHg (De Moudt et al., 2017), and aortic rings were equilibrated during 1 h to this preload prior to the start of the experiment, to ensure stable baseline conditions. After equilibration, preload was never externally adjusted. Isometric contractions and relaxations were measured by means of a Statham UC2 force transducer (Gould, United States). Contractions were induced by concentration-response stimulation with α_1_-adrenergic agonist phenylephrine (PE, 3 nM to 10 µM). Subsequently, voltage-gated calcium channels (VGCC) were blocked with 35 µM diltiazem to assess the contribution of VGCC to PE-induced contractions. Endothelium-dependent and–independent relaxations were determined in PE-precontracted aortic rings by concentration-response stimulation with acetylcholine (ACh, 3 nM to 1 µM) and diethylamine NONOate (DEANO, 0.3 nM-10 µM), respectively. For DEANO-induced relaxations, 300 μM L-NAME was added to exclude endogenous NO production. Finally, transient SR-mediated contractions were studied in a Krebs solution lacking calcium (0Ca Krebs) to avoid extra cellular calcium influx as previously described (Fransen et al., 2015). All concentration-response curves were fitted with a non-linear 4-parameter equation, to obtain values for maximal effect and half-maximal effective or inhibitory concentration (EC_50_ or IC_50_).

### Isobaric Measurement of Aortic Stiffness

2-mm aortic rings were mounted in a Rodent Oscillatory Tension set-up for Arterial Compliance (ROTSAC), between two parallel wire hooks in a 10-ml organ bath containing Krebs-Ringer solution. The upper wire hook was connected to a force-length transducer, and segments were continuously stretched between alternating preloads corresponding to calculated “systolic” and “diastolic” transmural pressures at a physiological frequency of 10 Hz to mimic the physiological heart rate in mice (600 bpm) as previously described (Leloup et al., 2016). At any given pressure, calibration of the upper hook allowed for the calculation of the diastolic and systolic vessel diameter (mm) and Peterson modulus (E_p_). E_p_ was defined as the pulse pressure divided by the relative diameter change (E_p_ = D_0_*ΔP/ΔD), and can be interpreted as the pressure change that is required to increase aortic diameter by 100%. Aortic stiffness was always assessed in isobaric conditions, and measured at oscillating calculated pressures of 60–100, 80–120, 100–140 and 120–160 mmHg. Contraction and relaxation of vessel segments were elicited as described above to assess different players in active contraction-dependent aortic stiffening.

### Histology

Aortic and cardiac tissue were fixed for 24 h in 4% formaldehyde solution (BDH Prolabo, VWR, Belgium), and subsequently dehydrated in 60% isopropanol (BDH Prolabo, VWR, Belgium), followed by paraffin-embedding. Aortic media thickness was measured on orcein-stained sections of the aorta, which allows for accurate assessment of the inner and outer border of the media layer. Total number of VSMC was ascertained by automated counting of the nuclei in the aortic media layer on fluorescent DAPI staining. Collagen composition of the media was ascertained by immunohistochemical staining with rabbit polyclonal anti-mouse collagen I (Abcam, ab21286), rabbit polyclonal anti-mouse/rat/cow/human collagen III (Abcam, ab7778), and rabbit polyclonal anti-mammal collagen IV (abcam, ab6586) antibodies. Collagen content was calculated as percentage area positivity in the region of interest (*i.e.*, the medial layer). Cardiac hypertrophy was quantified on the cellular level by rabbit polyclonal anti-mouse laminin (Novus Biologicals, nb300-144) staining of cardiac sections to measure myocardial cross-sectional area. For this measurement five images were recorded for each mouse in different cross-sectional regions of the heart and 20 cardiomyocytes were measured per image, in total averaging 100 cardiomyocyte measurements to obtain a final result. Microscopic images were acquired with universal Grap 6.1 software using an Olympus BX4 microscope and quantified using ImageJ software.

### Western Blot

The suprarenal abdominal aorta was lysed in Laemmli sample buffer (Bio-Rad) containing 5% β-mercaptoethanol. Samples were heat-denatured for 5 min and loaded on Bolt 4%–12% Gels (Life Technologies). After gel electrophoresis, proteins were transferred to Immobilon-FL membranes (Merck Millipore) according to standard procedures and incubated for 1 h in Odyssey Blocking Buffer (LI-COR Biosciences). Next, membranes were incubated at 4°C overnight with the following primary antibodies: rabbit polyclonal anti-mouse/rat/human eNOS (BD Biosciences, 610,299), mouse monoclonal anti-mouse/human p-eNOS (S^1177^) (BD Biosciences, 612,392), and mouse monoclonal anti-mouse/rat/human β-actin (Abcam, Ab8226). Finally, membranes were incubated with fluorescently labeled secondary antibodies (LI-COR Biosciences, anti-rabbit: IgG926-3221 and anti-mouse: IgG926-68070) to allow IR-detection and quantification on an Odyssey SA instrument (LI-COR Biosciences).

### Statistical Analysis

All data are expressed as mean ± SEM, with n representing the number of biological replicates. All analyses were performed using GraphPad Prism (version 8, GraphPad Software, San Diego, CA) and a significance level of 5% was set to identify statistically significant changes. Normality of data was verified using the Kolmogorov-Smirnov test, and parametric testing was used when indicated. This includes, one-way ANOVA, two-way ANOVA, three-way ANOVA, or multiple t-testing as indicated in the figure legends. A Tukey multiple testing correction was employed in post-hoc testing of the ANOVA tests.

## Results

### L-NAME Treatment Does not Affect Survival of C57Bl/6 Mice

**FIGURE 1:**
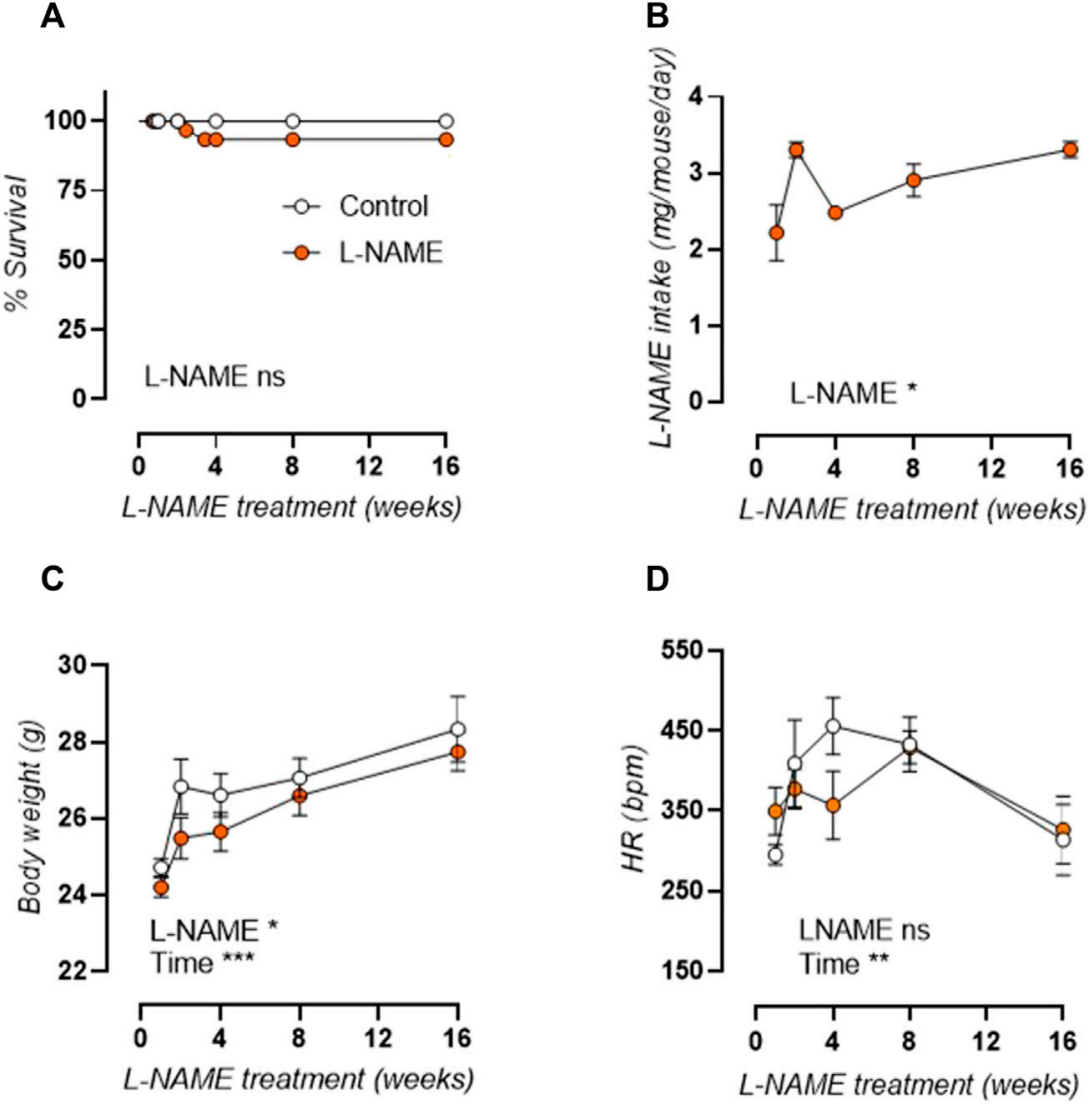
Overall parameters of longitudinal L-NAME treated C57Bl/6 mice. Survival curve **(A)**, L-NAME intake **(B)**, body weight **(C)**, and conscious heart rate **(D)** are shown for L-NAME treated (n ≥ 7) and control (n ≥ 9) mice. Statistical analysis was performed by Mantel-Cox log-rank test **(A)**, one-way ANOVA **(B)**, or two-way ANOVA **(C,D)**. Overall significance is listed at the bottom of the graph. *p < 0.05, **p < 0.01, ***p < 0.001.

General parameters of L-NAME treated and control mice are displayed in Figure 1. Survival analysis (Figure 1A) was unaffected by L-NAME treatment. Overall L-NAME intake (Figure 1B) showed significant variation between treatment times. Body weight was slightly reduced overall (Figure 1C) and conscious heart rate was unaltered (Figure 1D) in L-NAME treated mice.

### L-NAME Treatment Results in Fast-Onset Aortic Stiffness Prior to Blood Pressure Alterations and Cardiac Hypertrophy

**FIGURE 2:**
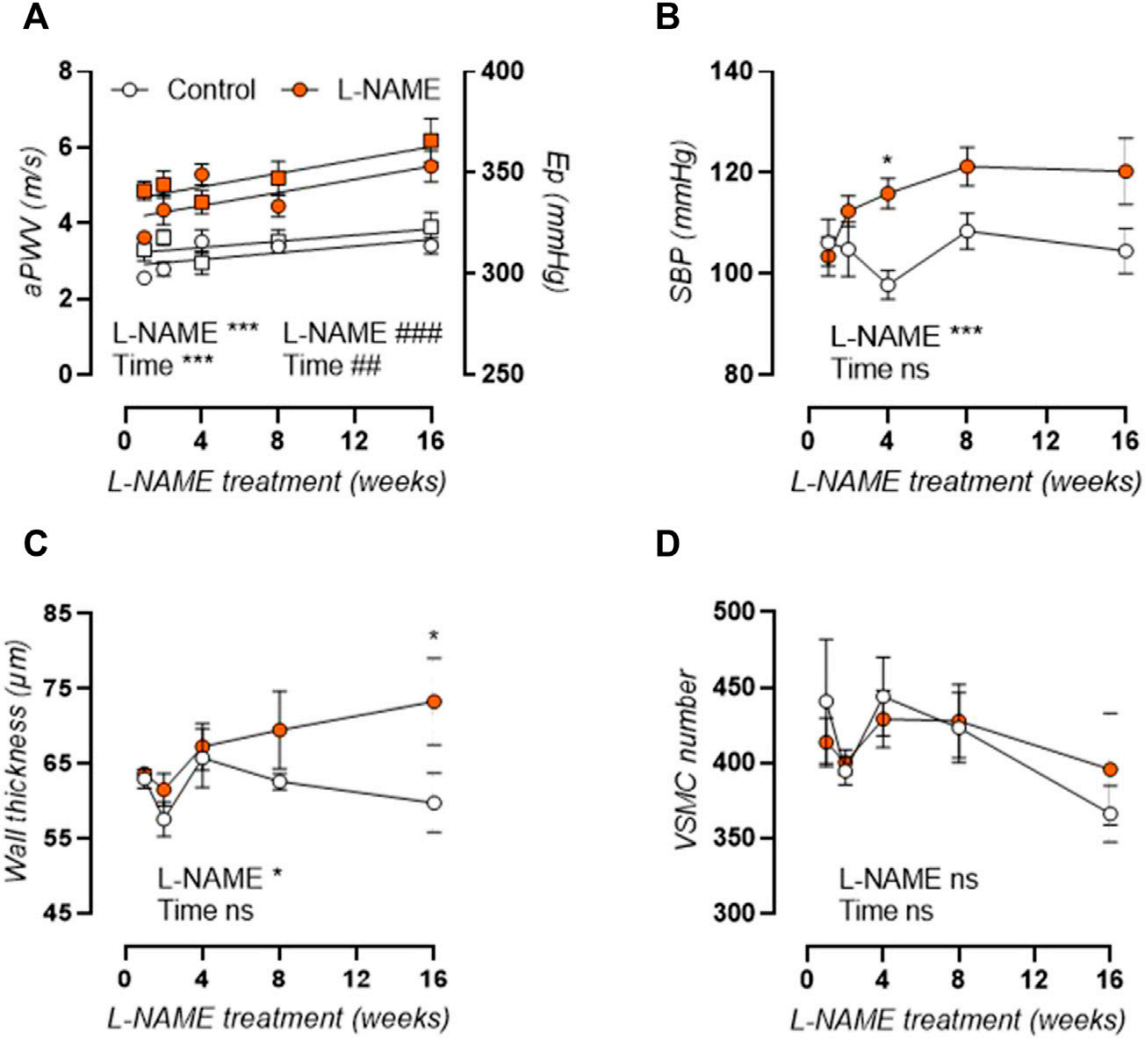
Aortic stiffness develops prior to peripheral hypertension in L-NAME treated C57Bl/6 mice. Aortic PWV (aPWV, circles, left *Y*-axis) and *ex vivo* Peterson modulus (E_p_, squares, right *Y*-axis) at isobaric 80–120 mmHg distending pressure **(A)** and systolic blood pressure (SBP, **(B)**) are shown for longitudinal L-NAME treated (n ≥ 7) and control (n ≥ 9) mice. Histology (n = 5) was employed to assess medial wall thickness **(C)** and VSMC number **(D)**. Linear regression is shown on for the aortic stiffness (aPWV and E_p_)-treatment duration relationship in A. Statistical analysis using two-way ANOVA. Overall significance (bottom) and post-hoc significance (in graph) are listed. Post-hoc significance was not listed in A. *p < 0.05, **p < 0.01, ***p < 0.001. ##*p* < 0.01, ###*p* < 0.001 for parameter E_p_ in A.

Measurement of *in vivo* aortic pulse wave velocity (aPWV, Figure 2A, left axis) and *ex vivo* Peterson modulus (E_p_, Figure 2A, right axis) showed increased aortic stiffness in L-NAME treated mice, which remained elevated over time. For aPWV, linear regression revealed an average + 0.0437 m/s and +0.0867 m/s slope of aortic stiffness-treatment duration relationship in control and L-NAME treated mice, respectively. For E_p_, this slope was +0.761 mmHg and +1.652 mmHg, respectively. L-NAME treatment thus doubled the slope in the aortic stiffness (aPWV and E_p_)-treatment duration relationship, suggesting a two times higher rate of stiffening in L-NAME treated mice.

**FIGURE 3:**
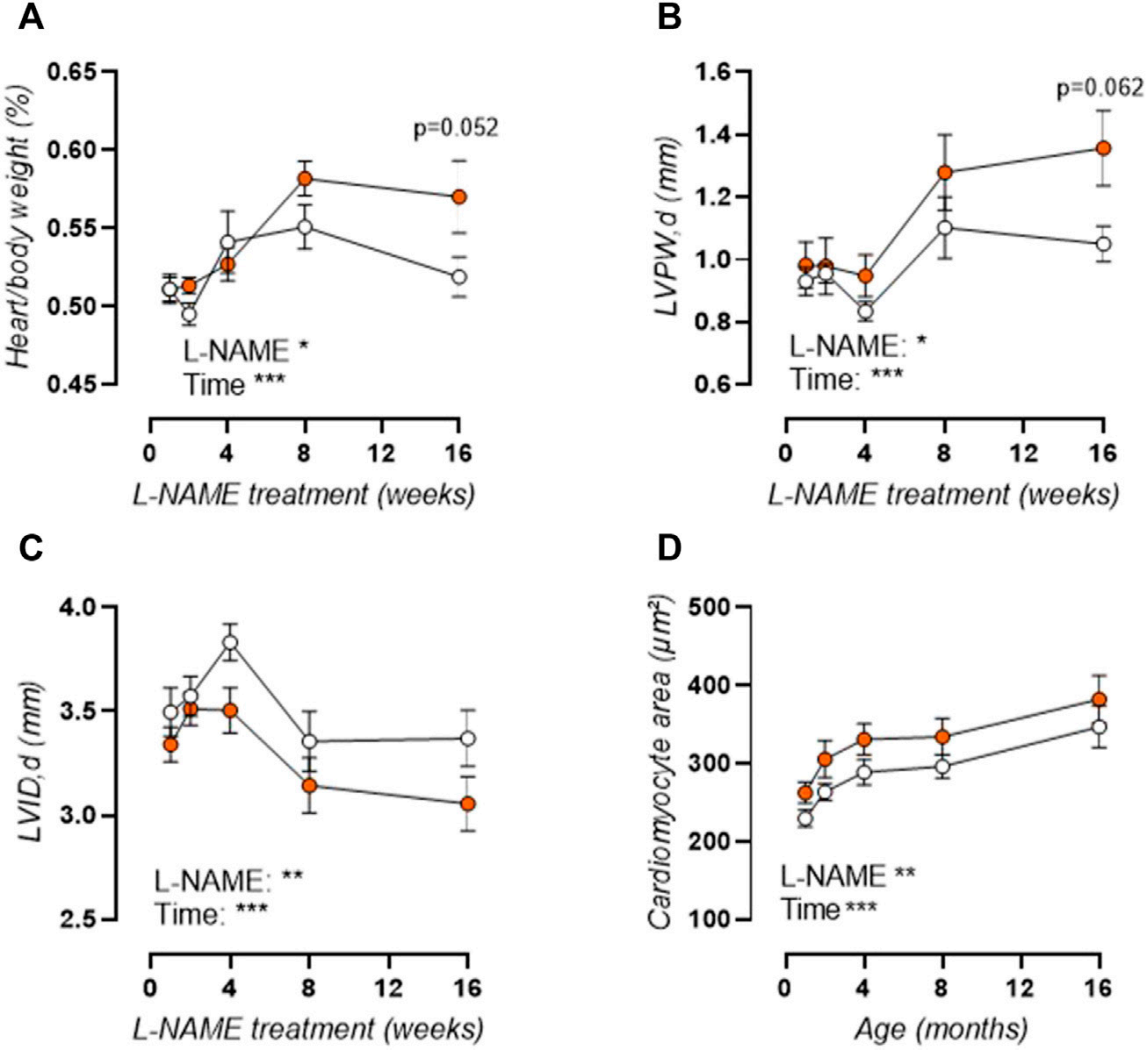
L-NAME induced cardiac hypertrophy. Heart over body weight ratio **(A)** and echocardiographic assessment of left-ventricular posterior wall (LVPW) thickness **(B)** and left-ventricular inner diameter (LVID, **(C)**) were assessed in L-NAME treated (n ≥ 7) and control (n ≥ 9) mice, and were combined with immunohistochemical anti-laminin staining for measurement of cardiomyocyte cross-sectional area (n = 5) **(D)** to assess cardiac hypertrophy. Statistical analysis using two-way ANOVA. Overall significance (bottom) and post-hoc significance (in graph) are listed. *p < 0.05, **p < 0.01, ***p < 0.001.

As such, aortic stiffening preceded peripheral hypertension which developed progressively after 4 weeks treatment, with increased systolic (Figure 2B), diastolic (not shown), and mean (not shown) blood pressure. Furthermore, histological analysis revealed a late-term increase in aortic wall thickness (Figure 2C), without a change in VSMC number (Figure 2D), implying VSMC hypertrophy. Associated cardiac hypertrophy developed only after 4–8 weeks of L-NAME treatment and was characterized by increased heart/body weight (Figure 3A), left-ventricular posterior wall thickness (Figure 3B), and decreased left-ventricular lumen diameter (Figure 3C), indicating concentric cardiac hypertrophy. Hypertrophy was confirmed on the cellular level by histological measurement, but showed a constant rather than progressive increase due to L-NAME treatment (Figure 3D) (representative image, Supplementary Figure S1). Ejection fraction and diastolic left ventricular relaxations were preserved throughout the L-NAME treatment (data not shown).

### L-NAME Treatment Attenuates the E_p_-Pressure Relation

**FIGURE 4:**
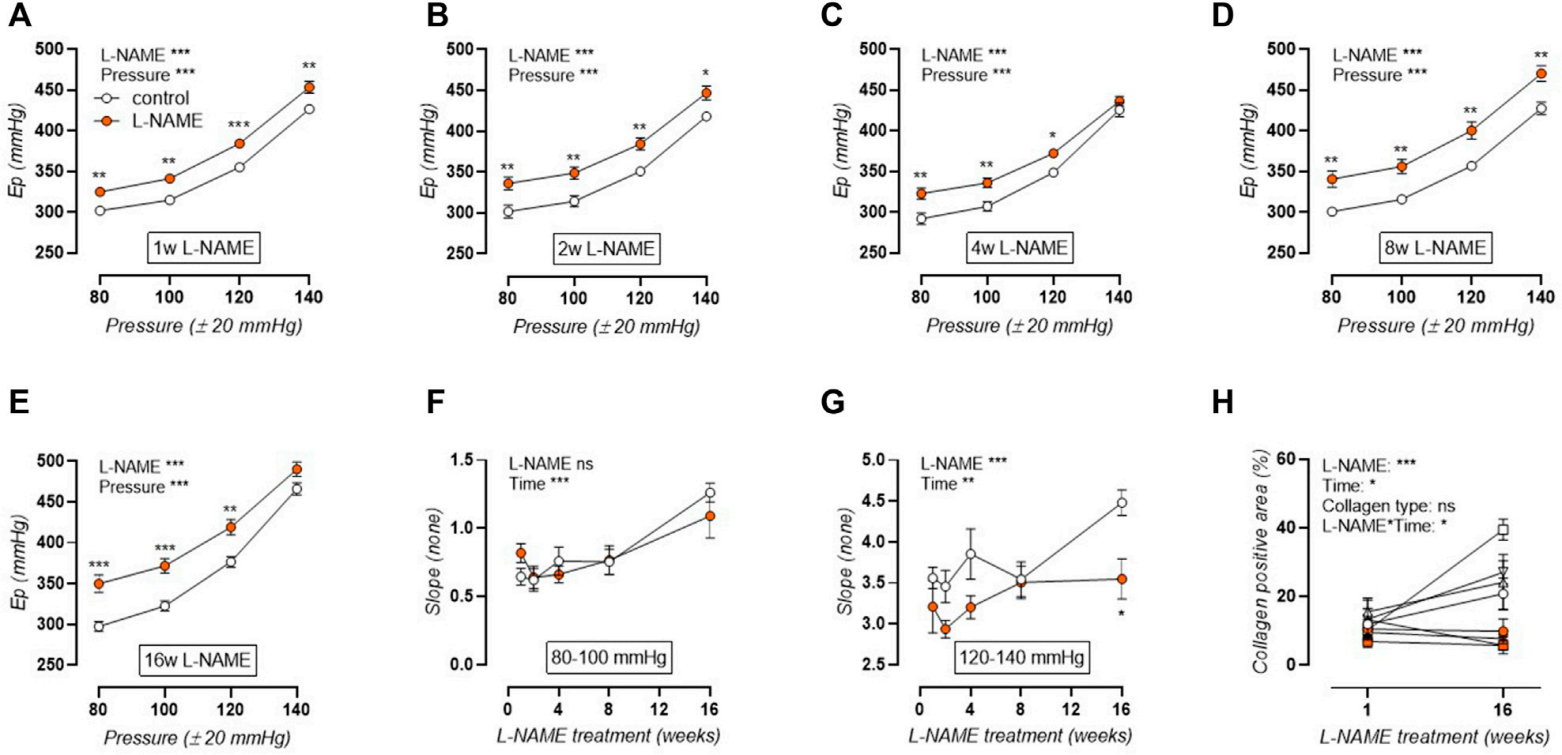
Aortic stiffness-pressure relation is attenuated by L-NAME treatment. Peterson modulus (E_p_)-pressure curves are shown for increasing treatment durations: 1 week **(A)**, 2 weeks **(B)**, 4 weeks **(C)**, 8 weeks **(D)** and 16 weeks **(E)** L-NAME treated (n ≥ 7) and control (n ≥ 9) mice. The slope of the lower (mean pressure 80 mmHg to mean pressure 100 mmHg, **(F)**) and upper (mean pressure 120 mmHg to mean pressure 140 mmHg, **(G)**) range of the E_p_-curve was calculated and plotted longitudinally. Collagen composition was determined using immunohistochemical staining (n = 5) for collagen I (circles, **(H)**), collagen III (squares, **(H)**), and collagen IV (upward triangle, **(H)**). The average value for collagens I, III, and IV was included in the graph (downward triangle, **(H)**), but was not included in the statistical analysis. Statistical analysis using two-way ANOVA **(A–G)** or three-way ANOVA **(H)**. Overall significance (top) and post-hoc significance (in graph) are listed. No post-hoc significance is listed in H. *p < 0.05, **p < 0.01, ***p < 0.001.

E_p_ was studied over a pressure range from calculated 60–100 to 120–160 mmHg distending pressures, revealing that aortic stiffness was increased over the entire pressure range and across all treatment durations (Figures 4A–E). Calculation of the slope of the E_p_-pressure relation in the lower pressure range, *i.e.*, mean pressure 80 mmHg to mean pressure 100 mmHg, revealed that this slope remained conserved after L-NAME treatment (Figure 4F). Contrarily, at the higher pressure range, *i.e.*, mean pressure 120 mmHg to mean pressure 140 mmHg), an overall attenuation of the E_p_-pressure slope was observed in L-NAME treated mice, which was most pronounced after 16-weeks treatment (Figure 4G). Since E_p_ at high distending pressure is mainly dependent on extracellular collagen deposition in baseline (uncontracted) conditions, medial collagen composition was investigated. Decreased medial collagen I, III, and IV positive area was observed after 16-weeks L-NAME treatment (Figure 4H) (representative image, Supplementary Figures S2-S4). Interestingly, contrary to control mice where a significant increase in collagen content was noted after 16 weeks, collagen content did not increase significantly over time in L-NAME treated mice.

### L-NAME Induced Endothelial Dysfunction is Transient

**FIGURE 5:**
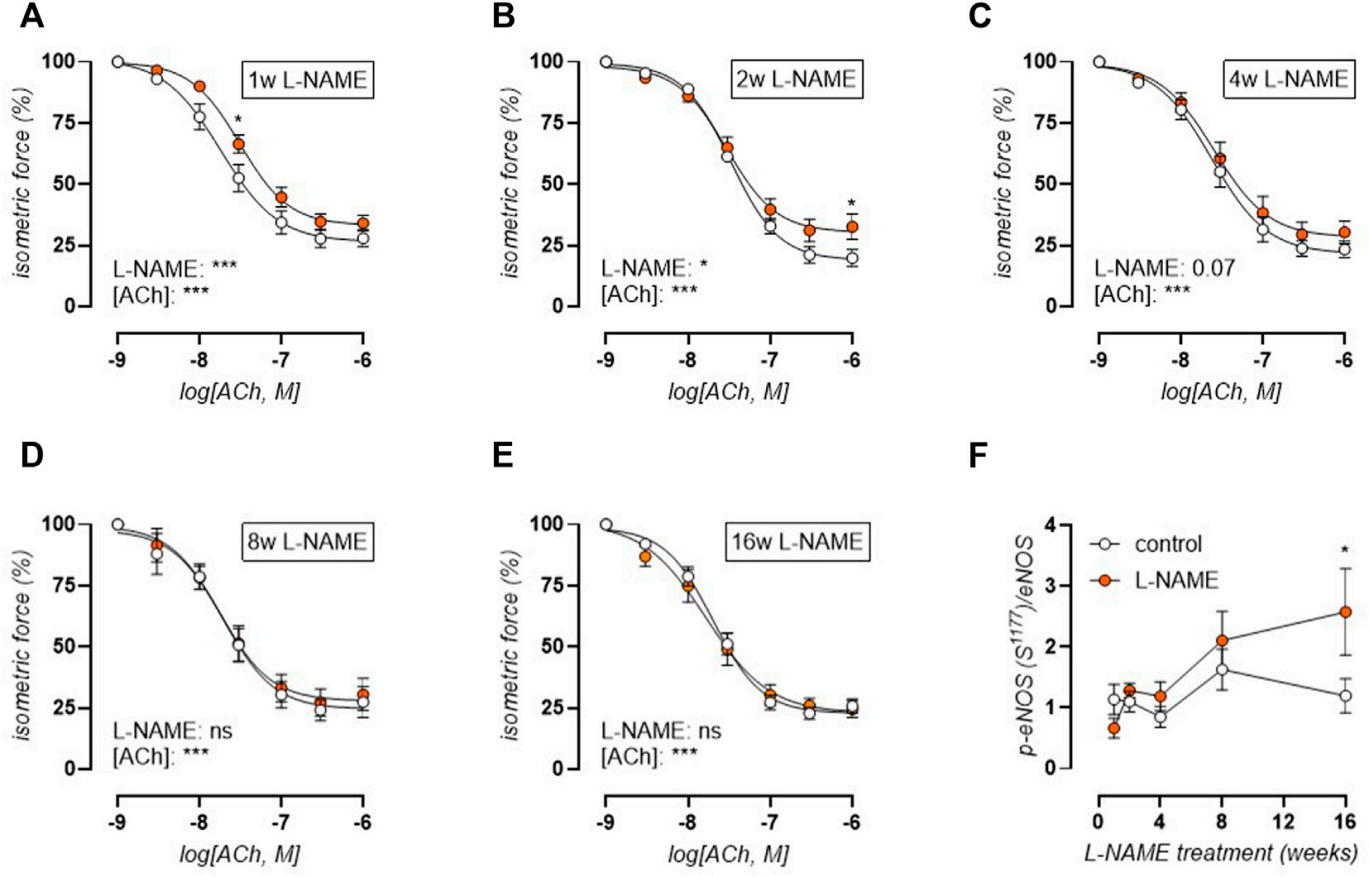
Acetylcholine (ACh) relaxations reveal early endothelial dysfunction and late-term normalization. Concentration-response curves are shown for increasing treatment durations: 1 week **(A)**, 2 weeks **(B)**, 4 weeks **(C)**, 8 weeks **(D)**, and 16 weeks **(E)** L-NAME treated (n ≥ 7) and control (n ≥ 9) mice. Western blot analysis (n = 5) of phospho-eNOS (S^1177^)/eNOS **(F)**. Statistical analysis using two-way ANOVA (**(A-E)**, overall significance: bottom, post-hoc significance: in graph) or multiple t-tests (**(F)**, significance in graph). *p < 0.05, **p < 0.01, ***p < 0.001.

Acetylcholine (ACh) concentration-response stimulation of 2 µM PE-precontracted aortic rings was used to confirm L-NAME induced endothelial dysfunction. Indeed, short-term L-NAME treatment (1–4 weeks) resulted in impaired ACh-induced relaxations (Figures 5A–C). After 1 week of L-NAME treatment, ACh-induced relaxation impairment was most pronounced, with a significant right shift of the curve (IC_50_, log(M): 7.7 ± 0.1 control, -7.5 ± 0.1 L-NAME, *). Endothelial dysfunction was attenuated over time, reaching complete restoration by 8 weeks of treatment (Figure 5D). Western blot analysis of eNOS (S^1177^) phosphorylation level revealed an early non-significant reduction (*p* = 0.40) in L-NAME treated mice. From 2 weeks onward however, p-eNOS (S^1177^) increased, reaching statistical significance after 16 weeks treatment, indicating that the restoration of endothelial function might result from compensatory eNOS activation by S^1177^ phosphorylation (Figure 5F) (representative image, Supplementary Figure S5). Relaxations induced by exogenous NO donor DEANO were unchanged (Supplementary Figure S6), confirming that the above-mentioned changes in ACh-induced relaxations were due to altered endothelial NO function.

**FIGURE 6:**
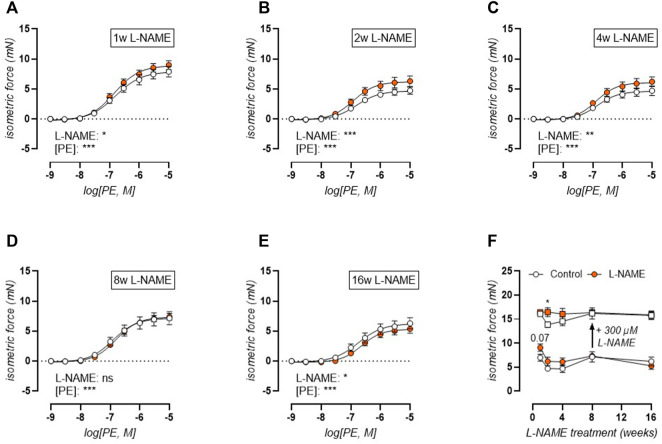
Phenylephrine (PE) contractions are heightened in the early phase with late-term reduction. Concentration-response curves are shown for increasing treatment durations: 1 week **(A)**, 2 weeks **(B)**, 4 weeks **(C)**, 8 weeks **(D)**, and 16 weeks **(E)** L-NAME treated (n ≥ 7) and control (n ≥ 9) mice. Maximal effect of the non-linear regression analysis (circles, **(F)**) and maximal PE-induced contractions after inhibition of basal NO production by 300 μM L-NAME (squares, **(F)**) were plotted longitudinally. Statistical analysis using two-way ANOVA (**(A-E)**, overall significance: bottom) or multiple t-tests (**(F)**, separate analyses for circles and squares, significance in graph). *p < 0.05, **p < 0.01, ***p < 0.001.

We have previously shown that the mouse aorta releases significant basal NO, which has a strong attenuating effect on PE-induced contractions (Leloup A. J. A. et al., 2015). Therefore, the concentration-contraction relationship was studied during the treatment period from 1 to 16 weeks (Figure 6). Short-term (1–4 weeks) L-NAME treated mice displayed heightened contractions to phenylephrine (PE, Figures 6A–C) stimulation, whereas 8-weeks L-NAME treatment resulted in a normalization of PE-induced contractions (Figure 6D), and even a reduction after 16 weeks treatment (Figure 6E). A maximal PE-induced contraction was elicited by subsequent addition of 300 μM L-NAME to inhibit basal NO production (Figure 6F). This showed that after 1-week L-NAME treatment, no difference in maximal α_1_-adrenoreceptor-dependent contractility could be observed, meaning that the increased PE-induced contraction in the absence of L-NAME resulted from impaired basal NO production. After 2–4 weeks L-NAME treatment, increased PE-induced contractions remained in the presence of 300 μM L-NAME, indicating increased α_1_-adrenoreceptor-dependent contractility. From 8 weeks L-NAME treatment onward, no difference in α_1_-adrenoreceptor-dependent contractility was observed.

### Late-Term Shift Towards VSMC Dysfunction

**FIGURE 7:**
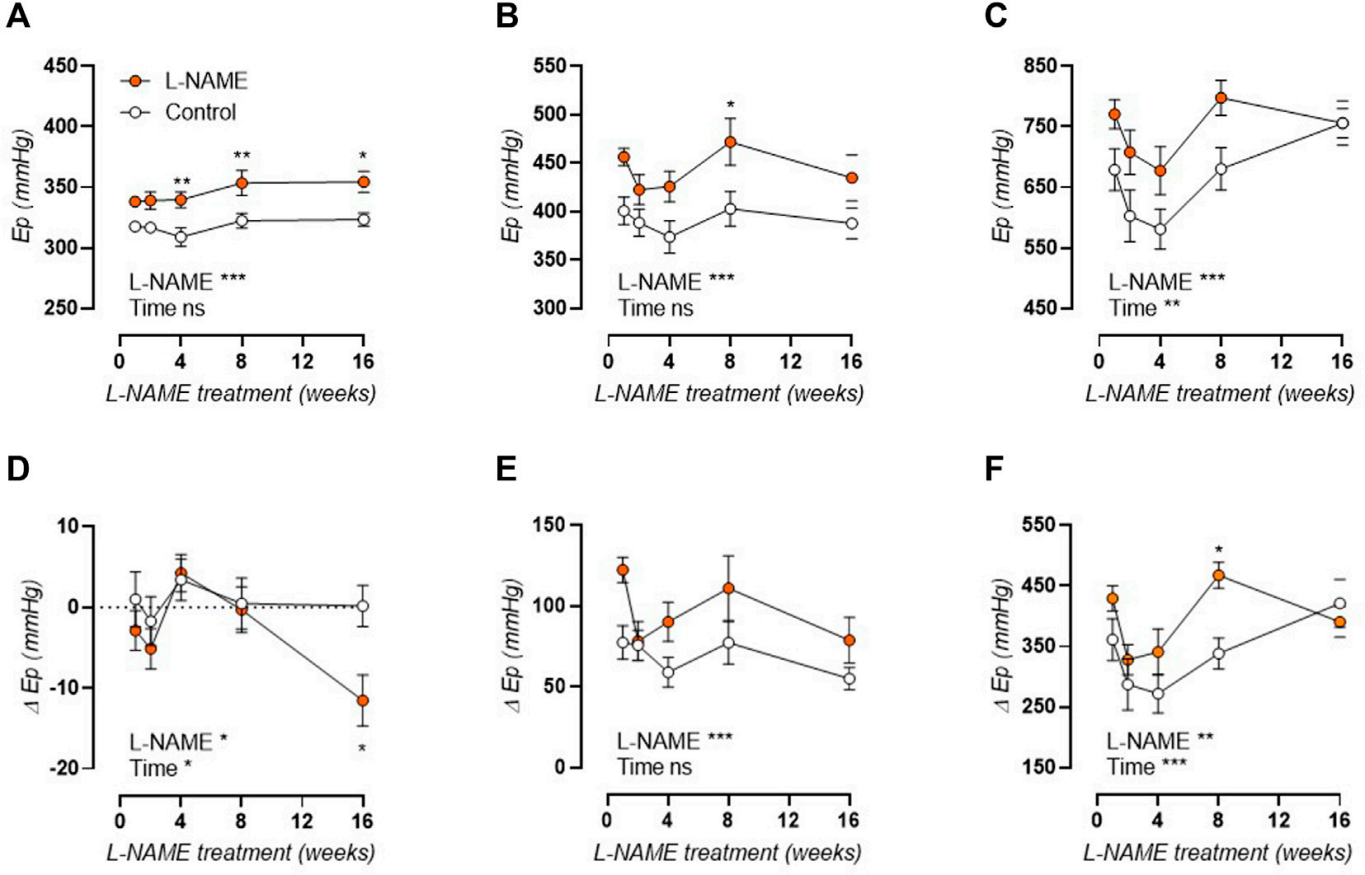
Contraction independent and dependent aortic stiffness in longitudinal L-NAME treated mice. Peterson modulus (E_p_) at isobaric 80–120 mmHg distending pressure is shown in the absence of extracellular calcium (0Ca, **(A)**), in the presence of 2 μM PE **(B)**, and in the presence of 2 μM PE and 300 μM L-NAME **(C)** in L-NAME treated (n ≥ 7) and control (n ≥ 9) mice. Stiffness in these conditions was further compared to baseline E_p_ values in Krebs-Ringer solution to obtain the effect of extracellular calcium removal **(D)**, 2 μM PE contraction **(E)**, and 2 μM PE with 300 μM L-NAME contraction **(F)** on aortic stiffness. Statistical analysis using two-way ANOVA. Overall significance (bottom) and post-hoc significance (in graph) are listed. *p < 0.05, **p < 0.01, ***p < 0.001.

Biomechanical testing of the isolated thoracic aorta was employed to test contraction-independent and -dependent aortic stiffness. Contraction-independent aortic stiffness was ascertained in a Krebs-Ringer solution lacking extracellular calcium (0Ca) to abolish all contractile tone (Figure 7A). This revealed continuously elevated isobaric aortic stiffness in L-NAME treated mice. Moreover, when comparing E_p_ in 0Ca Krebs to baseline values, a late-term drop in stiffness was observed after 16-weeks L-NAME treatment, indicating a measurable VSMC contractile tone after long-term treatment (Figure 7D). In the presence of 2 μM PE, aortic stiffness increased due to active VSMC contraction in both control and L-NAME treated mice (Figure 7B). Subsequent inhibition of basal NO production by 300 μM L-NAME, resulted in further contraction-dependent aortic stiffening, and revealed increased E_p_ in L-NAME treated mice at all time-points other than after 16 weeks, where E_p_ no longer differed from control values (Figure 7C). The active contraction-dependent stiffening was calculated as E_p_ in contracted conditions minus baseline E_p_ in Krebs-Ringer solution, and revealed increased PE-induced aortic stiffening in L-NAME treated mice (Figure 7E). In the presence of L-NAME, contraction-dependent stiffening was increased in 1–8 weeks L-NAME treated mice, but showed a decrease after 16-weeks treatment (Figure 7F).

**FIGURE 8:**
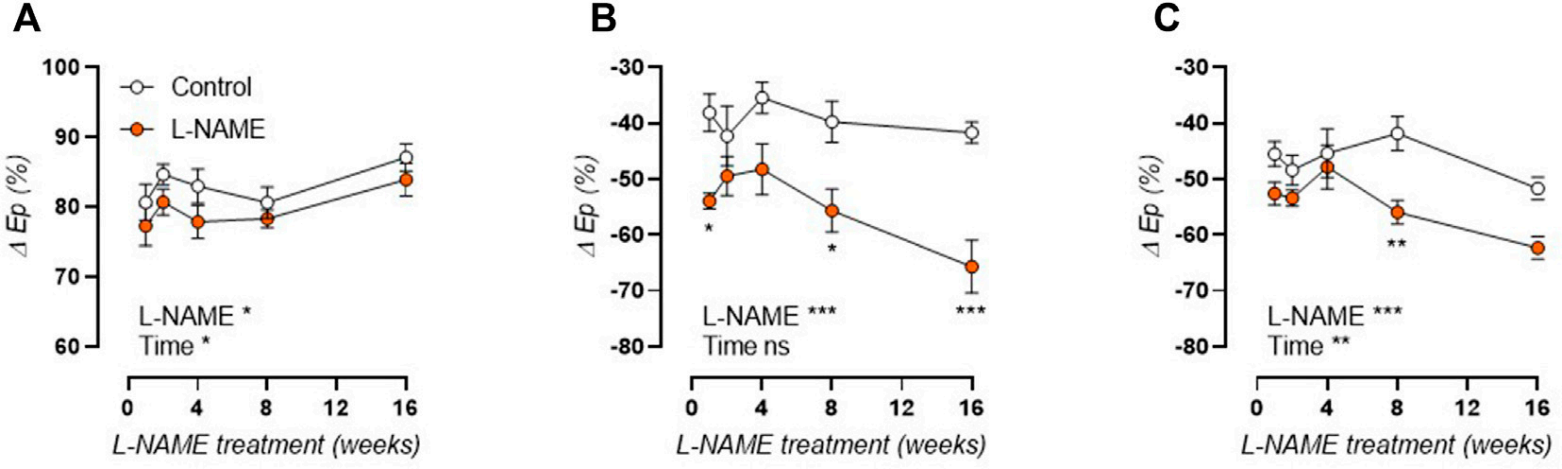
Reduced basal NO levels and increased voltage-gated calcium channel contribution in L-NAME treated mice. Basal NO levels were calculated as the relative difference in E_p_ (80–120 mmHg distending pressure) by 2 μM PE in the absence and presence of NOS blocker L-NAME (300 μM, **(A)**) in L-NAME treated (n ≥ 7) and control (n ≥ 9) mice. The relative effect of 35 µM diltiazem on PE-induced E_p_ increase was used to calculate the contribution of VGCC to contraction-dependent stiffening in the absence **(B)** and presence **(C)** of NOS blocker L-NAME (300 µM) in L-NAME treated (n ≥ 7) and control (n ≥ 9) mice. Statistical analysis using two-way ANOVA. Overall significance (bottom) and post-hoc significance (in graph) are listed.*p < 0.05, **p < 0.01, ***p < 0.001.

Furthermore, biomechanical testing revealed a constantly reduced effect of basal NO levels on the aortic stiffness of L-NAME treated mice, ascertained as the relative difference in contraction-dependent stiffening by 2 μM PE in the absence and presence of NOS blocker L-NAME (300 μM, Figure 8A). The contribution of voltage-gated calcium channels to α_1_-adrenergic contraction-dependent stiffening was assessed through diltiazem (35 µM)-mediated relaxation of 2 µM PE-precontracted aortic rings. A pattern of early increase in the acute phase, and an even more pronounced increase in the late-disease phase was revealed (Figure 8B). In the absence of basal NO production (addition of 300 μM L-NAME), a similar pattern of increased VGCC contribution remained even though NOS-inhibition only induced an increase in VGCC contribution in control mice (Figure 8C).

### L-NAME Treatment Results in Heightened SR-Mediated Contractions

SR-mediated phasic contractions were induced by 2 μM PE in the absence of extracellular calcium, to avoid extracellular calcium influx. This phasic contraction was measured in the absence and presence of 300 μM L-NAME to investigate the importance of basal NO release on SR-mediated contractions (tracings of all SR-mediated contractions are available in Supplementary Figures S7, S8). The area under the curve (AUC), amplitude of the contraction phase (A_on_), and time constant of the relaxation phase (τ_off_) of the SR-mediated contractions are summarized in Table 1. A distinct reduction of the AUC of the SR-mediated contractions was observed by the addition of 300 μM L-NAME in both control and L-NAME treated mice. This was mostly due to an increased rate of calcium efflux (approximately 10 s reduction τ_off_). Contrarily, SR-mediated contractions of L-NAME-treated mice displayed an increased AUC value, both in the presence and absence of 300 μM L-NAME, indicating that chronic L-NAME treatment resulted in higher SR contractile calcium stores by affecting both intracellular calcium release (increased A_on_) and calcium removal (increased τ_off_). The increase in SR-mediated contractions in L-NAME treated mice was most pronounced in the early phase (1–4 weeks), followed by a normalization of SR-mediated contractions, which is most pronounced in baseline conditions (*i.e.*, absence of 300 μM L-NAME).

**TABLE 1:** Chronic L-NAME treatment results in heightened SR-mediated contractions.

	L-NAME (weeks)	Baseline				+ L-NAME (300 µM)			
		Control	L-NAME	*p*-Value LN	*p*-Value time	Control	L-NAME	*p*-Value LN	*p*-Value time
AUC (mN.s)	1	218.3 ± 18.9	265.6 ± 19.8	0.0016 (**)	0.0193 (*)	160.6 ± 20.4	188.1 ± 10.9	0.0172 (*)	0.2398
	2	169.0 ± 5.3	274.6 ± 33.8			152.0 ± 7.6	184.4 ± 20.6		
	4	172.4 ± 16.7	197.0 ± 14.4			141.7 ± 12.9	170.6 ± 26.4		
	8	218.6 ± 15.5	239.0 ± 23.5			166.4 ± 18.2	181.9 ± 19.0		
	16	182.3 ± 10.3	197.4 ± 26.5			119.2 ± 12.5	156.8 ± 24.2		
A_on_ (mN)	1	4.36 ± 0.18	4.76 ± 0.25	0.0014 (**)	0.0253 (*)	4.59 ± 0.33	5.59 ± 0.35	0.0020 (**)	0.0004 (***)
	2	4.39 ± 0.27	6.00 ± 0.42			6.68 ± 0.45	6.90 ± 0.35		
	4	4.58 ± 0.38	5.73 ± 0.57			5.26 ± 0.40	6.39 ± 0.68		
	8	5.29 ± 0.44	5.36 ± 0.27			4.90 ± 0.19	6.14 ± 0.36		
	16	5.44 ± 0.22	5.89 ± 0.25			6.23 ± 0.30	6.75 ± 0.6		
τ_off_ (s)	1	39.3 ± 4.9	46.0 ± 3.4	0.0600	<0.0001 (***)	23.5 ± 1.6	25.5 ± 1.1	0.0286 (*)	<0.0001 (***)
	2	22.5 ± 3.4	26.3 ± 3.1			11.4 ± 0.4	16.0 ± 1.5		
	4	16.6 ± 1.3	20.6 ± 1.3			13.4 ± 1.2	16.5 ± 1.3		
	8	26.9 ± 2.3	29.4 ± 4.0			19.0 ± 1.2	18.2 ± 1.8		
	16	21.2 ± 1.1	24.1 ± 4.1			14.3 ± 1.3	15.2 ± 1.2		

Values are expressed as mean ± SEM., Magnitude of the SR-mediated contractions was assessed as area under the curve (AUC) in the absence and presence of 300 μM L-NAME., Bi-exponential non-linear regression analysis was used to assess the amplitude of the contraction phase (A_on_) and time constant of the relaxation phase (τ_off_) in L-NAME, treated (n ≥ 7) and control (n ≥ 9) mice. Statistical analysis using two-way ANOVA., Overall significance is listed. *p < 0.05, **p < 0.01, ***p < 0.001.

## Discussion

The present study shows that L-NAME treatment induced fast-onset aortic stiffness (1 week treatment), which preceded the development of associated peripheral hypertension and cardiac hypertrophy (4–8 weeks treatment). L-NAME treatment-induced CV dysfunction was governed by three distinct temporal aortic disease phases: 1) an acute phase, 1–4 weeks treatment, characterized by impaired ACh-dependent aortic relaxations and heightened PE-induced contractions, 2) a pseudo-normalization phase, 8-weeks treatment, with restored vasoreactivity, and 3) a late-disease phase, 16-weeks treatment, with marked VSMC dysfunction features such as increased baseline contractile tone and heightened VGCC contribution to contraction-dependent aortic stiffening. This indicates that early L-NAME treatment-induced endothelial dysfunction shifts to late-term VSMC dysfunction.

### Arterial Stiffening Precedes Hypertension and Cardiac Hypertrophy in L-NAME Treated Mice

There has long been a chicken-and-egg debate regarding hypertension and arterial stiffness, since arterial stiffening is often regarded as an adaptive response to increased distending pressure, but has also been shown to precede hypertension in experimental models (Alderton et al., 2001; Weisbrod et al., 2013). Furthermore, epidemiological findings suggest that elevated aortic stiffness can predict future blood pressure changes whereas blood pressure has no predictive value for future PWV (Kaess et al., 2012). In the current study, increased arterial stiffness (both as *in vivo* aPWV and *ex vivo* E_p_) was observed after 1 week of L-NAME treatment, whereas peripheral blood pressure increased progressively but only became statistically significant after 4 weeks of L-NAME treatment. A progressive increase in blood pressure after initiation of L-NAME treatment is confirmed by independent studies (Boe et al., 2013; Li et al., 2016). For aPWV and E_p_, a significant increase is observed over time in both control and L-NAME treated mice. In part, this is an aging effect, since aortic stiffness increases significantly in mice from 9-weeks of age (after 1-week treatment) to 24-weeks of age (after 16-weeks treatment), as previously reported (Steppan et al., 2019; De Moudt et al., 2020). However, the slope of the aortic stiffness (aPWV or E_p_)-treatment duration relationship is twice as high in L-NAME treated vs. control mice, indicating that L-NAME treatment increases the rate of aortic stiffening. Cardiac hypertrophy, a well-known consequence of L-NAME treatment (Paulis et al., 2008; Bunbupha et al., 2019; Soliman et al., 2019; Jing et al., 2021), developed progressively in L-NAME treated mice, reaching near-significance (*p* = 0.052 for heart/body weight, *p* = 0.062 for left ventricular posterior wall thickness) after 16-weeks treatment, although cellular hypertrophy was already observed after 1-week treatment and remained stably increased throughout the treatment period.

### Arterial Stiffening in L-NAME Treated Mice is Both Contraction-Dependent and -Independent

The current study demonstrates that aortic stiffness in L-NAME treated mice is increased in baseline conditions, with a pressure-dependent trend towards attenuation at high distending pressure. This attenuation of the contraction-independent aortic stiffening coincided with a late-onset decline in collagen content, including types I, III, and IV collagen, which might act as a compensatory mechanism to lessen aortic stiffness as progressive hypertension developed, since collagen is the main load-bearing extracellular matrix (ECM) component at high distending pressure (Hodis and Zamir, 2011; Safar, 2016). This is, however, in contrast with most CV aging models, where accumulation of collagen in the arterial media is observed with increasing age, aortic stiffness, and blood pressure (Hosoda et al., 1984; Andreotti et al., 1985; Tsamis et al., 2013). Furthermore, NO has been described to inhibit collagen production in VSMC *in vitro* (Kolpakov et al., 1995; Rizvi and Myers, 1997; Myers and Tanner, 1998), which is also in conflict with the observation of reduced collagen content after chronic NOS inhibition.

The present study describes a similar response of medial collagen types I, III, and IV to L-NAME induced aortic stiffening. In control C57Bl/6 mice, collagen content increased with age, whereas this increase was absent in L-NAME treated mice, leading to attenuated aortic stiffening in L-NAME treated mice at high distending pressure. Although this response was most pronounced for collagen type III, it was also detected for collagen types I and IV. Contrary to the fibril-forming collagen types I and III, for which the role in aortic biomechanics has been well-defined (Vouyouka et al., 2001; Holzapfel, 2008), type IV collagen is situated in the endothelial and VSMC basement membranes (Berillis, 2013) and is therefore a less likely candidate to be involved in arterial stiffness regulation. However, polymorphisms in the COL4A1 gene have been associated to PWV changes (Adi et al., 2015) and reduced collagen type IV crosslinking in peroxidasin-deficient mice was shown to decrease renal tubular basement membrane stiffness, indicating that the basement membrane may indeed possess biomechanical properties (Bhave et al., 2017). Also, VSMC phenotype was shown to be dependent on the extracellular substrate, with a collagen IV rich substrate leading to higher smooth muscle α-actin expression compared to a fibronectin substrate (Thyberg and Hultgårdh-Nilsson, 1994). Furthermore, increased myocardial type IV collagen expression was noted after induction of pressure overload left ventricular hypertrophy (Chapman et al., 1990), indicating that collagen type IV levels may respond to changes in pressure.

Aside from these contraction-independent ECM changes, aortic stiffening in L-NAME treated mice was also contraction-dependent, as evidenced by the heightened effect of α_1_-adrenoreceptor-dependent contraction on active aortic stiffening after stimulation with 2 μM PE, with or without additional inhibition of basal NO production by 300 μM L-NAME. The extent of α_1_-adrenoreceptor-dependent aortic stiffening normalized, however, after long-term (16-weeks) L-NAME treatment. This is confirmed by isometric reactivity studies, where concentration-response stimulation with PE elicited heightened contractions in the early phase (1–4 weeks) but not the late phase (8–16 weeks). Previous work by our research group has established the importance of VSMC contraction in the active regulation of aortic stiffness (Leloup et al., 2019), as confirmed by independent studies (Gao et al., 2014; Lacolley et al., 2017; Nolasco et al., 2020), indicating that heightened contractile behavior of the aorta of L-NAME treated mice might impair the active regulation of the aortic pressure-stiffness relationship.

### L-NAME Treatment Results in Transient Endothelial Dysfunction

In the present study, endothelial dysfunction was confirmed by isometric measurement of ACh-induced vasorelaxation in 2 µM PE-preconstricted aortic rings and by *ex vivo* biomechanical measurement of basal NO production (*i.e.*, the difference in contraction-dependent stiffening by 2 μM PE in the absence and presence of NOS blocker L-NAME). Although the results demonstrated constantly reduced basal NO production, impaired ACh-induced NO responses were only observed after short-term (1–4 weeks) but not long-term (8–16 weeks) L-NAME treatment. Although some studies describe the absence of endothelial dysfunction after chronic L-NAME treatment (Desai et al., 2006; Fitzgerald et al., 2007; Balis et al., 2010), others describe persistence of endothelial dysfunction (De Gennaro Colonna et al., 2005; Qu et al., 2010; Li et al., 2016; Berenyiova et al., 2018). These dissimilarities might be due to a difference in the studied animal model (*e.g.*, C57Bl/6 mice (Fitzgerald et al., 2007), Wistar rats (Paulis et al., 2008), spontaneously hypertensive [SHR] rats (Berenyiova et al., 2018)), treatment dose (from 0.3 to 100 mg/kg (Fitzgerald et al., 2007; Balis et al., 2010), ∼110 mg/kg in the current study), or treatment duration (from 5 to 10 weeks (Balis et al., 2010; Berenyiova et al., 2018)). Since L-NAME acts as a competitive non-specific inhibitor of the NOS enzymes, the restoration of endothelial function might be explained by any compensatory mechanism that enhances NOS function (*e.g.*, gene upregulation, phosphorylation, increased substrate or cofactor availability), that improves the sensitivity of the underlying VSMC to endothelial NO, or that activates NOS-independent vasorelaxant pathways (*e.g.*, endothelium-derived hyperpolarizing factor [EDHF], prostacyclin). In the present study, VSMC sensitivity to NO was unaltered in L-NAME treated mice, as assessed by concentration-response stimulation with exogenous NO-donor DEANO. Restoration of endothelial function coincided, however, with increased phosphorylation of eNOS at S^1177^, a well-known regulatory mechanism of eNOS function that promotes enhanced NO production (Fulton, 2016). Although not investigated in the present study, additional compensatory mechanisms ascribed to chronic L-NAME treatment in the scientific literature include increased cyclooxygenase (COX) activity and protein expression (Qu et al., 2010), activation of calcium-activated potassium channels through EDHF (Desai et al., 2006), and increased iNOS protein expression (Li et al., 2016). Another argument for the occurrence of compensatory changes after chronic L-NAME treatment lies in the observation that after discontinuation of L-NAME treatment, (partially) persistent hypertension, left ventricular hypertrophy, inhibition of aortic NO synthase activity, and compensatory COX-2 expression have been observed (Paulis et al., 2008).

Furthermore, several other compensatory vasodilatory substances may contribute to the restoration of endothelial function. In this context, insulin-induced vasodilation may represent a potential player. Insulin can elicit vasorelaxation through activation of eNOS and/or through stimulation of EDHF production (Chen and Messina, 1996; Oltman et al., 2000; Iida et al., 2001). It was previously reported that insulin-mediated dilation of small coronary arteries of obese Zucker rats was impaired in the presence of preserved ACh-mediated vasodilation responses (Katakam et al., 2005). Similarly, insulin-induced forearm vasodilator responses were blunted in obese vs. lean human subjects, whereas ACh-induced vascular responses were similar between both populations (Tack et al., 1998). These studies indicate variant regulation of insulin- and ACh-mediated vasorelaxant responses. Additionally, neuregulin-1, a cardioprotective growth factor, was also shown to compensate for impaired NO production in the aorta of eNOS knockout mice (Shakeri et al., 2021), again demonstrating that alternative pathways may compensate for endothelial NO dysfunction.

### L-NAME Treatment Results in Aberrant VSMC Calcium Handling

VGCC represent the foremost calcium entry pathway in VSMC, illustrated by the pronounced phenotype of SMC-specific Cacna1C knockout mice (*i.e.,* disestablished myogenic tone and severe hypotension) (Moosmang et al., 2003). In the present study, the contribution of VGCC to α_1_-adrenoreceptor-mediated contraction-dependent aortic stiffening was slightly increased in the early phase (1 week L-NAME), then normalized (2–4 weeks L-NAME), followed by a marked progressive increase in the late-disease phase (8–16 weeks L-NAME). The effect in the early phase is most likely the result of VSMC depolarization due to endothelial dysfunction (Leloup A. J. et al., 2015). In the late phase, however, the increased VGCC activity indicates pathological ion channel remodeling due to chronic exposure to high arterial stiffness and hypertension (Harder et al., 1983). Increased VGCC activity in CV disease has been reported in cardiomyocytes (Navaratnam and Khatter, 1990; Wetzel et al., 1991) and VSMC of various arterial beds [*i.e.,* aorta (Harder et al., 1983; Hui et al., 1999), coronary arteries (Badin et al., 2018), mesenteric arteries (Chen et al., 2015)]. On the other hand, decreased VGCC expression and activity with CV disease were also reported (Fukuda et al., 2014), and the exact role of VGCC is thus up for debate. Interestingly, VGCC contribution was highly dependent on the presence of basal NO in control mice, since inhibition of NO production by addition of 300 μM L-NAME induced an approximately 20% increase in VGCC contribution, whereas this NO-dependence was entirely absent in L-NAME treated mice (all treatment durations). These findings again underline the different effects between chronic L-NAME treatment *in vivo* and acute exposure to L-NAME *ex vivo*.

Aside from the VGCC alterations, L-NAME treated mice also displayed increased SR-mediated contractions, indicating altered intracellular calcium handling which resulted in increased SR contractile calcium stores. L-NAME treated mice displayed larger release of contractile calcium from the SR stores and a slower rate of calcium removal from the cytoplasm. These functions are mainly dependent on the inositol 1,4,5-trisphosphate (IP3) receptor (ITPR) and plasma membrane calcium ATPase (PMCA), respectively, as previously described (Leloup A. J. et al., 2015), suggesting alterations in IP3R and PMCA function in L-NAME treated mice. Analogous to the findings regarding VGCC, it was striking that chronic L-NAME treatment had opposite effects on the SR-mediated contractions compared to acute inhibition of NO production by L-NAME *ex vivo*. Increased SR-mediated contractions were mostly observed in the early phase (1–4 weeks), after which these findings largely normalized. Interestingly, the normalization of SR-mediated contractions coincided with the restoration of normal ACh-induced vasorelaxations and with the onset of increased basal VSMC cytoplasmic calcium in long-term (8–16 weeks) L-NAME treated mice. Taken together, most of the changes in VSMC calcium signaling (*i.e.*, VGCC function and cytoplasmic calcium load) occurred after endothelial function had already normalized, demonstrating that reversal of the initial stimulus (*i.e.*, impaired NO production) was insufficient to impede disease phenotype progression in chronic L-NAME treated mice.

### Role of Endothelial Dysfunction in Cardiovascular Disease

The present study demonstrates that induction of endothelial dysfunction by L-NAME treatment induced a distinct CV disease phenotype, including progressive aortic stiffening, hypertension, and cardiac hypertrophy, as previously described (Boe et al., 2013; Li et al., 2016). Interestingly, the disease continued to progress even though the original stimulus (*i.e.*, impaired endothelial function) had normalized, which included continued progression of both *in vivo* (*e.g.*, aPWV, blood pressure, heart weight) and *ex vivo* (*e.g.*, VGCC contribution, basal VSMC cytoplasmic calcium loading) disease characteristics. To our best knowledge, we are the first research group to describe this distinct shift from early endothelial dysfunction to late-term VSMC dysfunction in chronic L-NAME treated C57Bl/6 mice. Endothelial dysfunction has long been denominated as a CV aging hallmark (Tao et al., 2004; Rossman et al., 2018; Majerczak et al., 2019). This is emphasized by the multitude and effectiveness of endothelial function targeting therapeutic interventions, including NO-releasing non-steroidal anti-inflammatory drugs (Fiorucci et al., 2001), statins (Laufs and Liao, 1998; Nakata et al., 2007; Zhang et al., 2012), hormone therapy (Kawano et al., 2003; Williams et al., 2004), resveratrol (Schmitt et al., 2010; Crandall et al., 2012), and dietary factors (Dickinson et al., 2009; Mccall et al., 2009; Blumenthal et al., 2010). Contrarily, there have been observations that arterial aging occurs independent of endothelial dysfunction (Del Campo et al., 2019; De Moudt et al., 2020), confirming that arterial disease can also occur predominantly at the VSMC level, as seen in the present study after long-term L-NAME treatment. Interestingly, improved NO function was demonstrated to ameliorate CV disease even in disease models where arterial aging occurred primarily at the VSMC level (Del Campo et al., 2019).

### Study Limitations

Previous work by our research group demonstrated the importance of isometric preload on aortic physiology (De Moudt et al., 2017). Therefore, isometric reactivity studies were performed at a 20-mN preload, corresponding to an approximately 100 mmHg mean distending pressure in a healthy adult mice (De Moudt et al., 2017). However, preload was not adjusted for age or L-NAME treatment in the present study. Post-hoc analysis of the *ex vivo* ROTSAC organ bath measurements showed that the ideal preload to obtain a 100 mmHg mean distending pressure was 22.7 mN for the present study, independent of age, but that a slightly lower preload should have been applied in L-NAME treated vs. control C57Bl/6 mice (illustrated in Supplementary Figure S9). However, the deviation from the applied 20 mN preload was not of the magnitude expected to affect isometric reactivity according to our previously published work (De Moudt et al., 2017) and was therefore not expected to greatly affect the data presented in this study.

The present study describes changes in medial collagen content, which coincided with attenuated aortic stiffening at high distending pressure. However, changes in three-dimensional collagen arrangement, potential ECM changes beyond collagen, or ECM-VSMC interactions were not considered in the present study, which might provide valuable insight in the role of ECM alterations in biomechanical aortic properties in arterial disease. Similarly, the present study describes important changes in calcium signaling pathways in VSMC of long-term L-NAME treated mice. Therefore, it would be interesting to further investigate the molecular targets underlying these changes in future research.

Furthermore, the present study only assessed L-NAME induced vasoactive alterations in aortic tissue, thereby overlooking the potential influence of peripheral arteries in L-NAME induced cardiovascular disease. Our research group previously demonstrated important differences in the physiology of elastic and resistance arteries (Leloup A. J. A. et al., 2015). Furthermore, we also demonstrated a variant role for elastic and muscular arteries in the pathophysiology of arterial disease in a context of autophagy deficiency (De Munck et al., 2020) and after short-term angiotensin-II treatment (Leloup et al., 2018), further highlighting the importance of both vessel types in the development of cardiovascular disease. Considering the importance of the peripheral arteries in blood pressure control, future studies on the time-dependent development of resistance artery vasoactive alterations may therefore greatly complement the findings in the present study, and improve the insight in the variant roles of elastic and resistance arteries in L-NAME induced cardiovascular disease.

Of note, all experiments presented in the present study were performed on separate cohorts of L-NAME treated mice at the end of their respective treatment durations, to avoid the influence of repeated measurements. For the non-invasive cardiovascular tests (*i.e.*, peripheral blood pressure, aPWV, and echocardiography), it would be interesting to include serial measurements in future studies to allow for intraindividual comparison. Furthermore, all experiments in the present study were performed in male C57Bl/6 mice, since previous studies showed that male rather than female C57Bl/6 mice exhibit key features of cardiovascular aging in humans (Sindler et al., 2011; Fleenor et al., 2013; Larocca et al., 2013). Sex differences in L-NAME induced cardiovascular disease were previously reported (Wang et al., 2003; Pavlov et al., 2009; Xue et al., 2009; Brinson et al., 2013). Therefore, the findings in the present manuscript should not be generalized to C57Bl/6 mice of both genders.

## Conclusion

The present study demonstrates that arterial stiffness precedes associated cardiovascular disease (*i.e.*, progressive peripheral hypertension and cardiac hypertrophy) in chronic L-NAME treated male C57Bl/6 mice. The underlying pathophysiological mechanisms of aortic aging consisted of three distinct phases: 1) an acute endothelial dysfunction phase (1–4 weeks L-NAME), 2) a pseudo-normalization phase with restored vasoreactivity (8 weeks L-NAME), and 3) a late-disease phase with aberrant VSMC function (16-weeks treatment). The study thus describes a unique shift from early endothelial dysfunction to late-term VSMC dysfunction, with continued cardiovascular disease progression despite normalization of the initial disease stimulus.

## Data Availability

The raw data supporting the conclusions of this article will be made available by the authors, without undue reservation.
