# Supplementary material for: Aortic Stiffness in L-NAME Treated C57Bl/6 Mice Displays a Shift From Early Endothelial Dysfunction to Late-Term Vascular Smooth Muscle Cell Dysfunction
Source: Front Physiol. 2022 Jun 16;13:874015. doi: 10.3389/fphys.2022.874015 (PMC9254682; doi:10.3389/fphys.2022.874015)
Supplement: Supplementary file 1 [file DataSheet1.pdf]

Supplementary Material

1 Supplementary Figures and Tables

1.1 Supplementary Figures

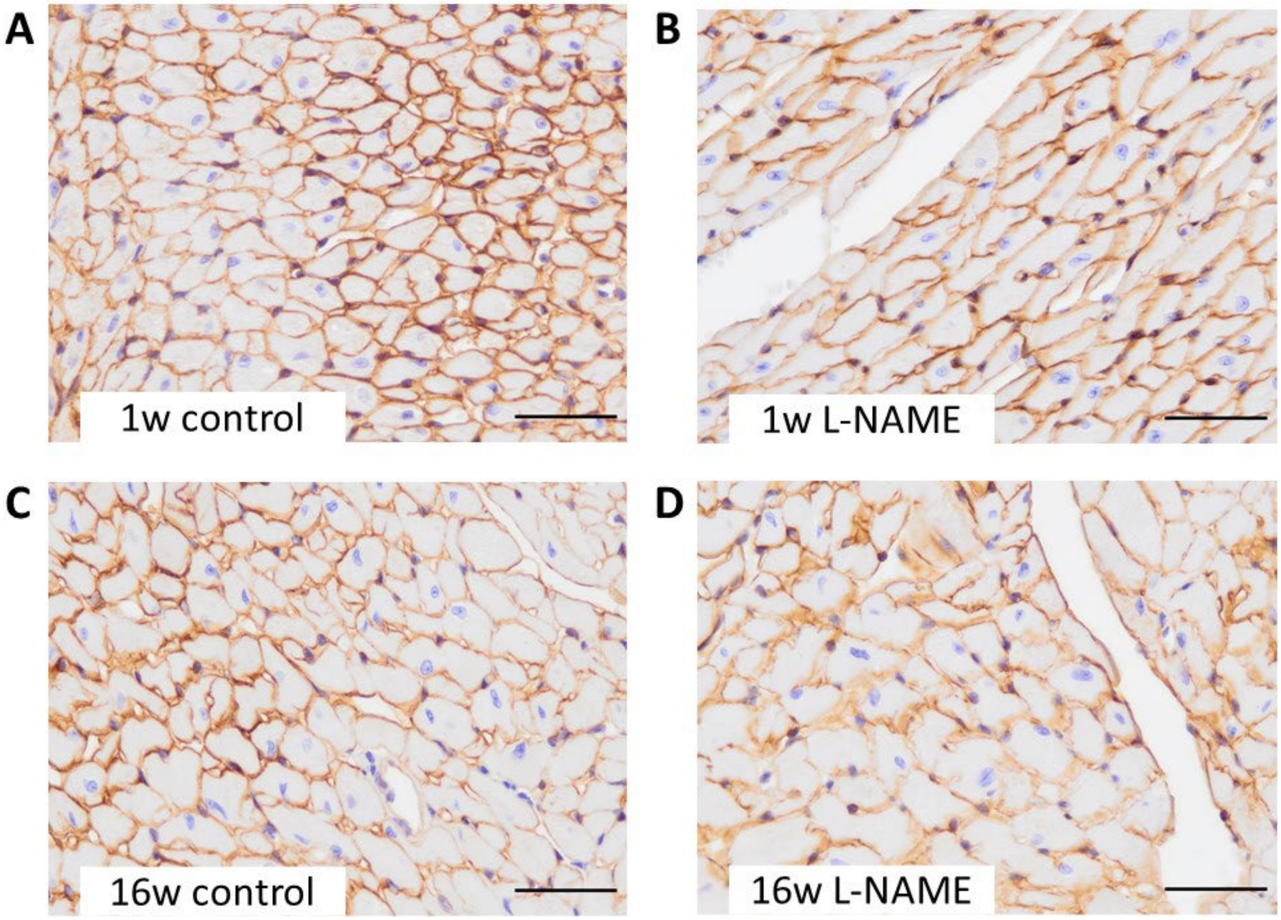

Supplementary Figure 1: Histological evaluation of cardiomyocyte cross-sectional area. Representative images of laminin-stained cardiac tissue of control (A, C) and L-NAME treated (B, D) mice after 1 and 16 weeks of treatment, respectively. Scale bars represent a length of 50 µm.

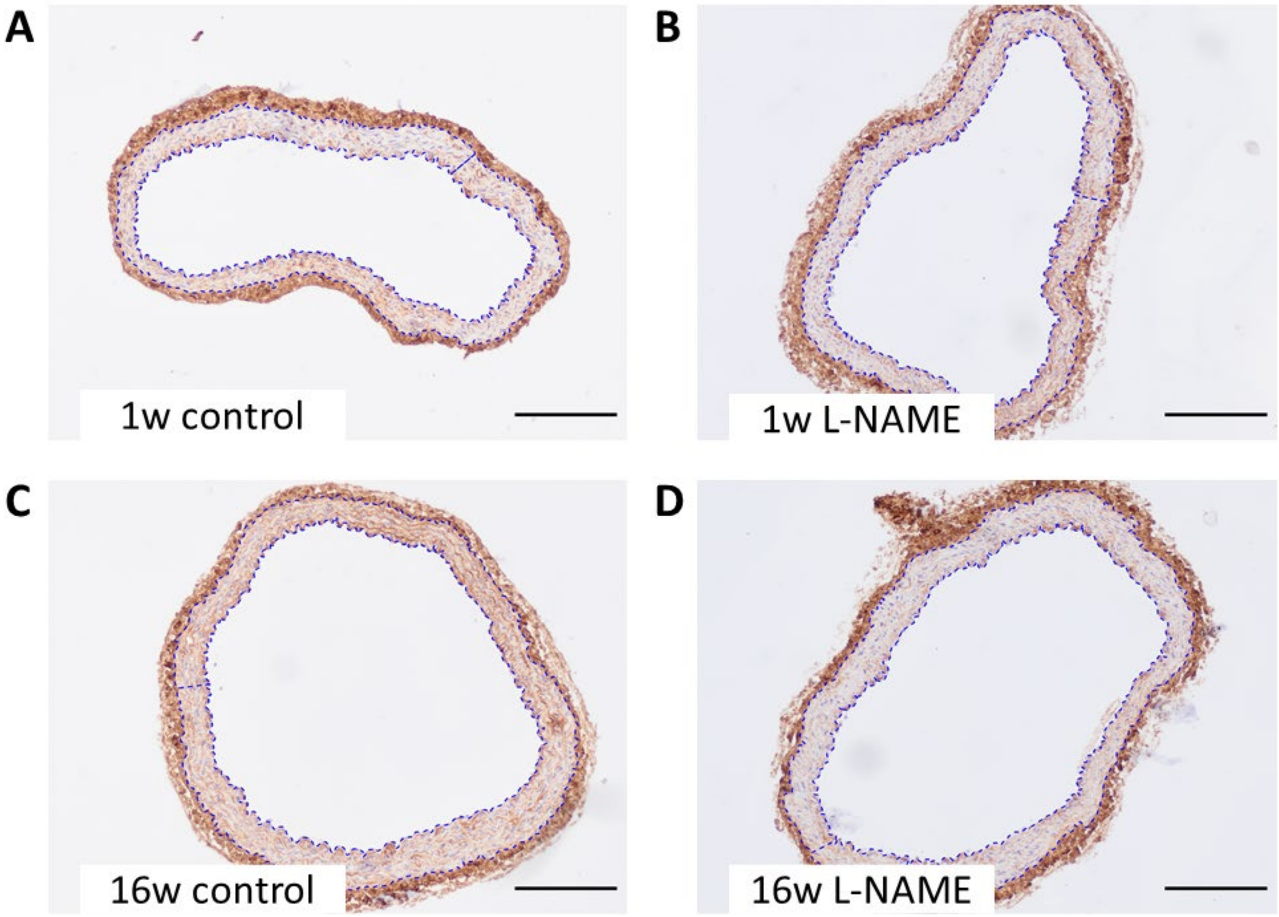

Supplementary Figure 2: Histological evaluation of aortic media collagen I content. Representative images of collagen I-stained aortic tissue of control (A, C) and L-NAME treated (B, D) mice after 1 and 16 weeks of treatment, respectively. Collagen I content was determined as the percentage area positivity in the region of interest (blue dotted line). Scale bars represent a length of 200 µm.

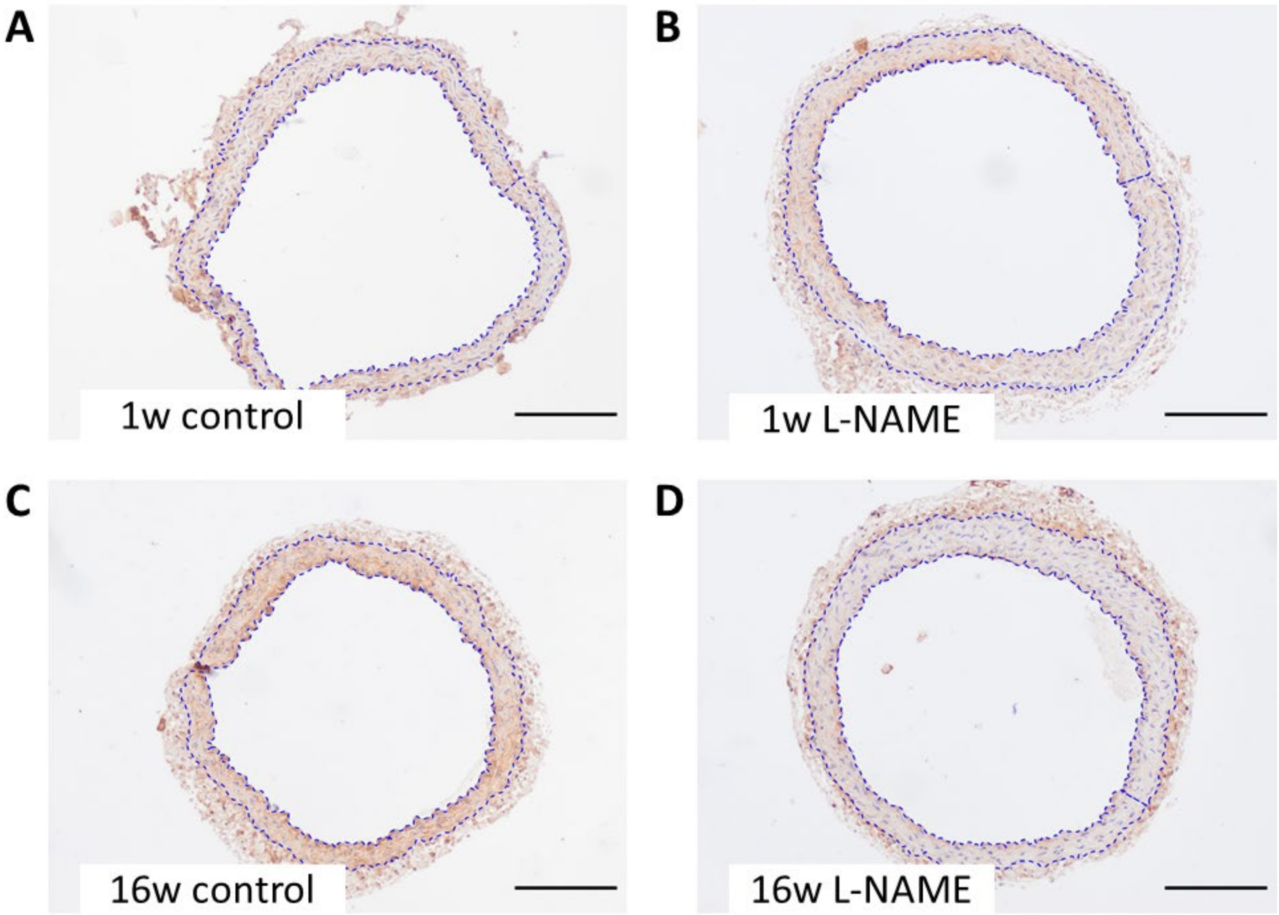

Supplementary Figure 3: Histological evaluation of aortic media collagen III content. Representative images of collagen III-stained aortic tissue of control (A, C) and L-NAME treated (B, D) mice after 1 and 16 weeks of treatment, respectively. Collagen III content was determined as the percentage area positivity in the region of interest (blue dotted line). Scale bars represent a length of 200 μm.

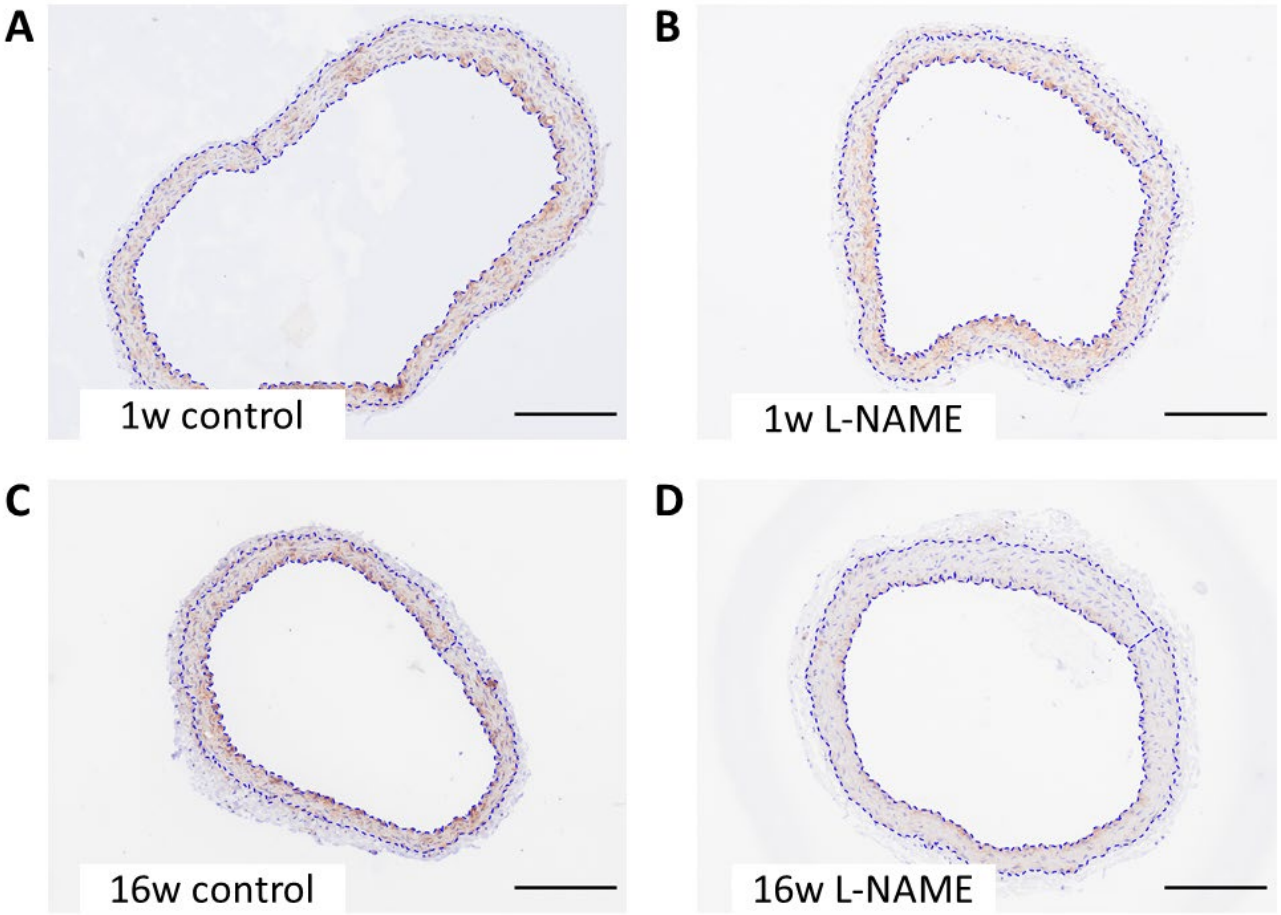

Supplementary Figure 4: Histological evaluation of aortic media collagen IV content. Representative images of collagen IV-stained aortic tissue of control (A, C) and L-NAME treated (B, D) mice after 1 and 16 weeks of treatment, respectively. Collagen IV content was determined as the percentage area positivity in the region of interest (blue dotted line). Scale bars represent a length of 200 µm.

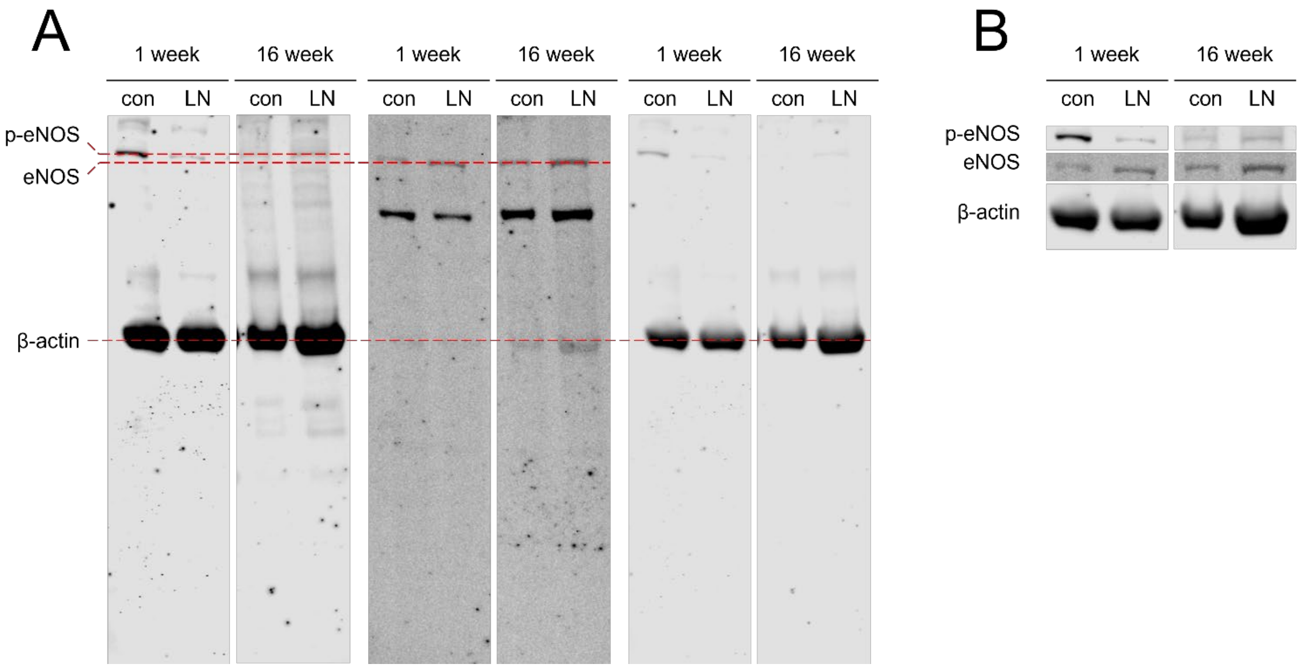

Supplementary Figure 5: Western blot evaluation of eNOS phosphorylation at S1177 in suprarenal abdominal aortic tissue lysates. Representative images of p-eNOS, eNOS, and β-actin protein bands of control (con) and L-NAME treated (LN) mice after 1 and 16 weeks of treatment are shown as full-length blots (A) or as summary overview (B). Western blots were recorded at 680RD (p-eNOS and β-actin) and 800CW (eNOS) IR channels. For quantification of p-eNOS and β-actin, Western blots were recorded using a different signal intensity.

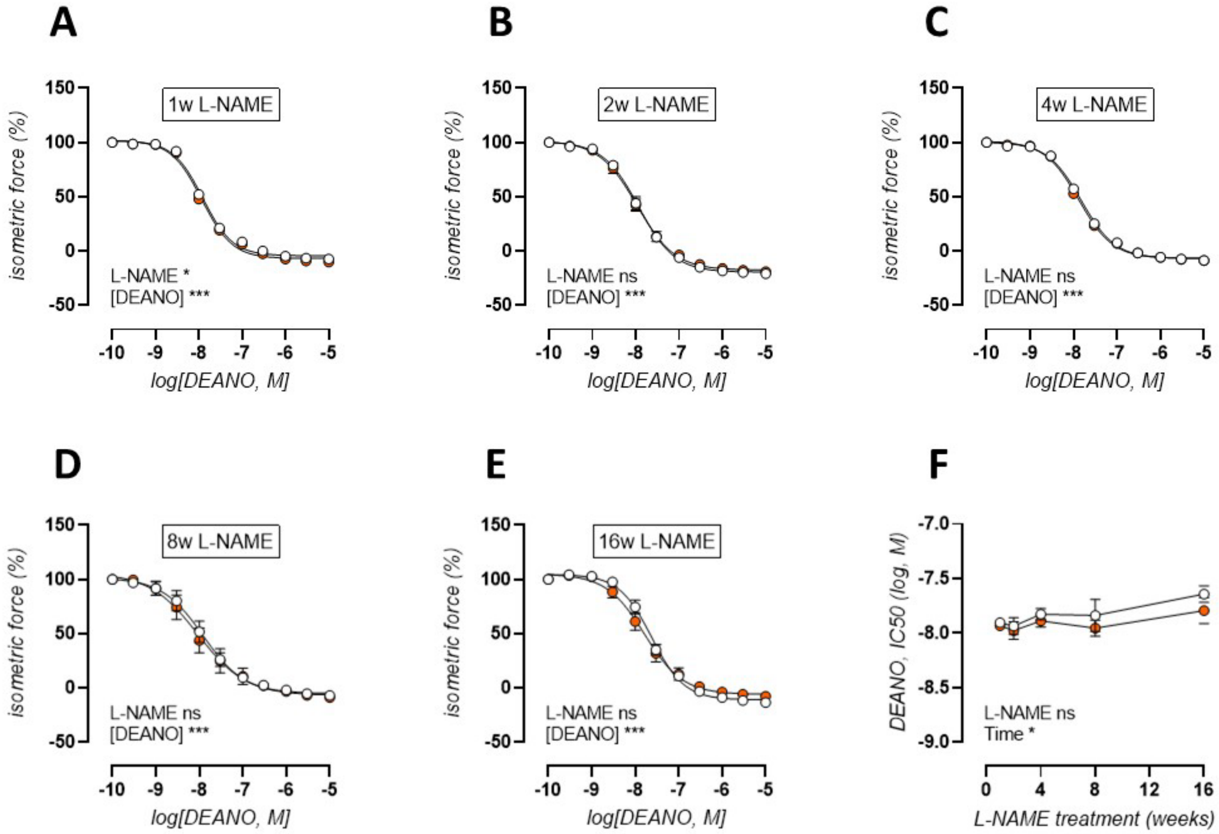

Supplementary Figure 6: Aortic relaxations to exogenous nitric oxide donor DEANO are unchanged. Concentration-response curves are shown for increasing treatment durations: 1 week (A), 2 weeks (B), 4 weeks (C), 8 weeks (D) and 16 weeks (E) L-NAME treated (n≥7) and control (n≥9) mice. IC_{50} of the non-linear regression analysis was plotted longitudinally (F). Statistical analysis using two-way ANOVA. Overall significance (bottom) and post-hoc significance (in graph) are listed.* $p<0.05$, *** $p<0.001$.

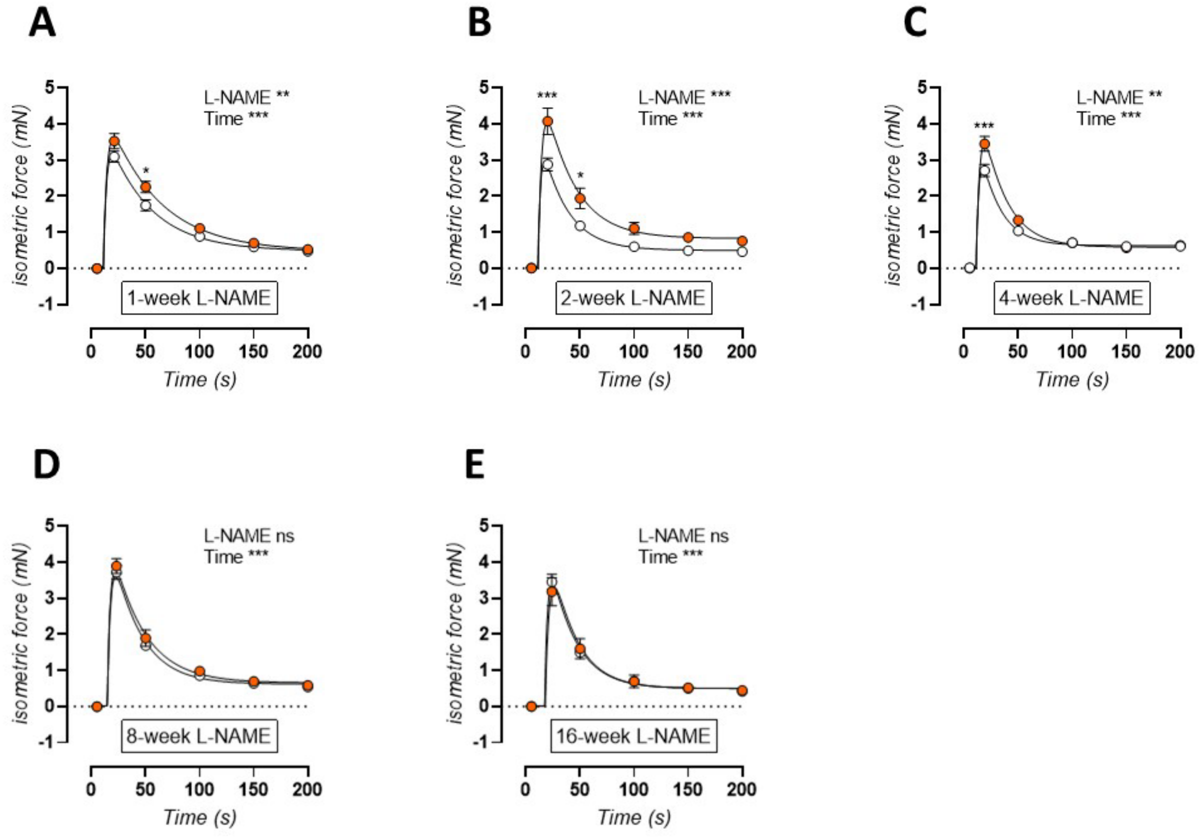

Supplementary Figure 7: Longitudinal SR-mediated contractions in the absence of NOS-blocker L-NAME. Transient SR-mediated contractions were induced by 2 µM PE in 0Ca Krebs, and tracings are shown for 1 week (A), 2 week (B), 4 week (C), 8 week (D), and 16 week (E) L-NAME treated (n≥7) and control (n≥9) mice. Statistical analysis using two-way ANOVA. Overall significance (top) and post-hoc significance (in graph) are listed.* $p<0.05$, ** $p<0.01$, *** $p<0.001$.

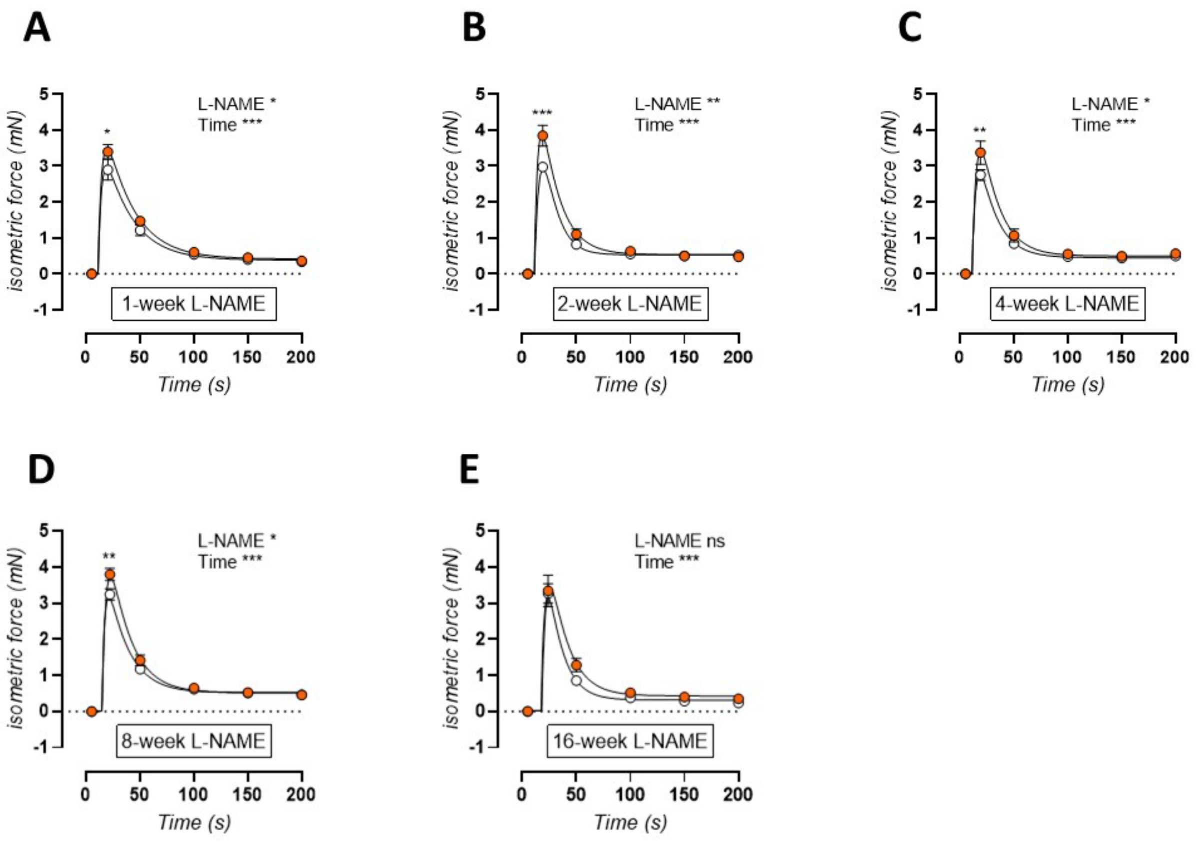

Supplementary Figure 8: Longitudinal SR-mediated contractions in the presence of NOS-blocker L-NAME. Transient SR-mediated contractions were induced by 2 µM PE in 0Ca Krebs supplemented by 300 µM L-NAME, and tracings are shown for 1 week (A), 2 week (B), 4 week (C), 8 week (D), and 16 week (E) L-NAME treated (n≥7) and control (n≥9) mice. Statistical analysis using two-way ANOVA. Overall significance (top) and post-hoc significance (in graph) are listed.* $p<0.05$, ** $p<0.01$, *** $p<0.001$.

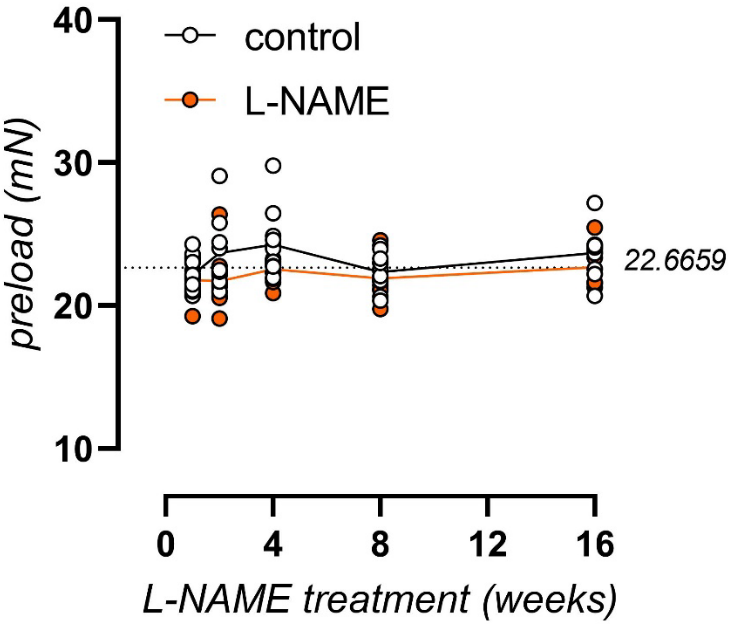

Supplementary Figure 9: Post-hoc analysis of the ideal preload to obtain a 100 mmHg distending pressure. From ROTSAC organ chamber experiments, the preload used to obtain a 100 mmHg distending pressure in baseline Krebs-Ringer conditions were plotted for L-NAME treated (n≥7) and control (n≥9) mice. On average, a 22.7 mN preload should have been applied for isometric reactivity studies.
